# Crystallization-Enhanced Emission and Room-Temperature Phosphorescence of Cyclic Triimidazole-Monohexyl Thiophene Derivatives

**DOI:** 10.3390/molecules28010140

**Published:** 2022-12-24

**Authors:** Daniele Malpicci, Alessandra Forni, Elena Cariati, Riku Inoguchi, Daniele Marinotto, Daniele Maver, Federico Turco, Elena Lucenti

**Affiliations:** 1 Department of Chemistry, Università degli Studi di Milano, Via Golgi 19, 20133 Milano, Italy; 2 Institute of Chemical Sciences and Technologies “Giulio Natta” (SCITEC) of CNR, Via Golgi 19, 20133 Milano, Italy; 3 INSTM Research Unit of Milano, Via Golgi 19, 20133 Milano, Italy; 4 Kyoto Institute of Technology, 1 Hashikami-cho, Matsugasaki, Sakyo-ku, Kyoto 606-8585, Japan

**Keywords:** organic room-temperature phosphorescence, crystallization-enhanced emission, nitrogen-rich emissive compounds

## Abstract

The development of organic room-temperature phosphorescent (ORTP) materials represents an active field of research due to their significant advantages with respect to their organometallic counterparts. Two cyclic triimidazole (**TT**) derivatives bearing one and three hexyl-thiophene moieties, **TT-HThio** and **TT-(HThio)_3_**, have been prepared and characterized. Both compounds display enhanced quantum yields in their crystalline form with respect to those in a solution state, revealing crystallization-enhanced emissive (CEE) behavior. Importantly, while single fluorescence is observed in solution, crystalline powders also feature dual ORTP, whose respective molecular and aggregate origins have been disclosed through X-ray diffraction analysis and DFT/TDDFT calculations. The relation between the photophysical properties of **TT-HThio** and its crystallinity degree has been confirmed by a decrease in photoluminescent quantum yield (Φ) and loss of vibronic resolution when its crystals are ground in a mortar, revealing mechanochromic behavior and confirming CEE features.

## 1. Introduction

Single-component organic materials characterized by rich emissive behavior, including room-temperature long-lived features, are receiving growing attention from the scientific community due to the benefits they offer with respect to their widely used metal-containing phosphorescent counterparts, including biocompatibility and low cost. Applications of organic room-temperature phosphorescence (ORTP) in different fields spanning bioimaging [1,2], anti-counterfeiting [3,4], catalysis [5] and displays [6] have been assessed. Many strategies, including π-π stacking interactions [7,8,9,10], host-guest systems [11,12,13], co-assembly based on macrocyclic compounds [14], crystallization [15,16] and cocrystallization [17], halogen bonding [18,19] and doping in a polymer matrix [20], have been developed to realize ORTP materials. In parallel, knowledge of the mechanisms involved in the photophysics of multi-emissive ORTP systems is evolving forward, step by step, aiming at the materials’ optimization.

In this context, in the last few years, our group has been involved in the preparation and characterization of various members of a family of compounds with triimidazo[1,2-*a*:1’,2’-*c*:1’’,2’’-*e*][1,3,5]triazine, **TT** [9], as a prototype. **TT** is characterized by crystallization-induced emissive (CIE) behavior, displaying, in particular, ultralong phosphorescence (ORTUP) (up to 1 s) under ambient conditions associated with the presence of strong π-π stacking interactions in its crystalline structure [7]. The presence of one or multiple heavy (Cl, Br and I) atoms or chromophoric fragments, namely 2-fluoropyridine, 2-pyridine or pyrene, on the **TT** scaffold greatly modifies both its molecular and solid-state photo-physical behavior, resulting in a complex, excitation-dependent photoluminescence with emissions comprising dual fluorescence, molecular phosphorescence and supramolecular ORTP and ORTUP [10,21,22,23,24,25,26,27]. Applications of **TT** derivatives in different fields including bio-imaging [26] and explosive detection [28] have been reported, and the identification of new members with different functionalities appears highly desirable. 

**Scheme 1:**
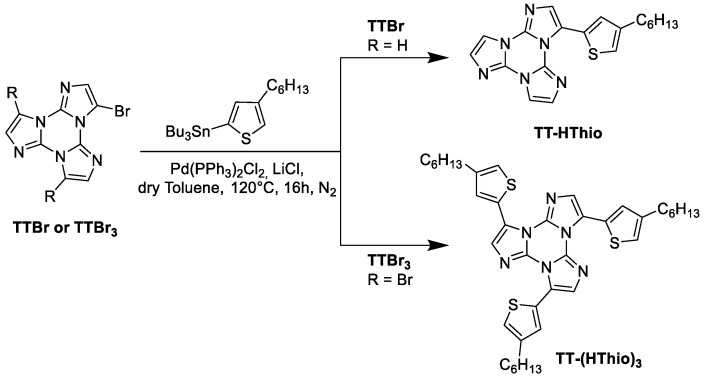
Synthesis of **TT-HThio** and **TT-(HThio)_3_**.

Herein, we describe the synthesis and structural and photophysical characterization of 3-(4-hexylthiophen-2-yl)-triimidazo[1,2-*a*:1’,2’-*c*:1”,2”-*e*][1,3,5]triazine, **TT-HThio**, and 3,7,11-tri(4-hexylthiophen-2-yl)-triimidazo[1,2-*a*:1’,2’-*c*:1”,2”-*e*][1,3,5]triazine, **TT-(HThio)_3_** (Scheme 1), with the hexylthiophene moiety being chosen for both its electron-donor properties and the ability to produce organic crystals that can respond to mechanical stimuli [29,30].

**TT-HThio** and **TT-(HThio)_3_** display enhanced properties in the crystalline form with respect to their solutions revealing CEE (crystallization-enhanced emissive) behavior. Importantly, while single fluorescence is observed in solution, crystals are also characterized by dual phosphorescence, whose origin has been disclosed through X-ray diffraction analysis and DFT/TDDFT calculations. 

## 2. Results

3-(4-hexylthiophen-2-yl)-triimidazo[1,2-*a*:1’,2’-*c*:1”,2”-*e*][1,3,5]triazine, **TT-HThio**, and 3,7,11-tri(4-hexylthiophen-2-yl)-triimidazo[1,2-*a*:1’,2’-*c*:1”,2”-*e*][1,3,5]triazine, **TT-(HThio)_3_**, have been synthetized by Stille coupling between tributyl(4-hexylthiophen-2-yl)stannane and the corresponding mono- (**TTBr**) or tri-brominated (**TTBr_3_**) **TT** derivative (Scheme 1).

**Figure 1:**
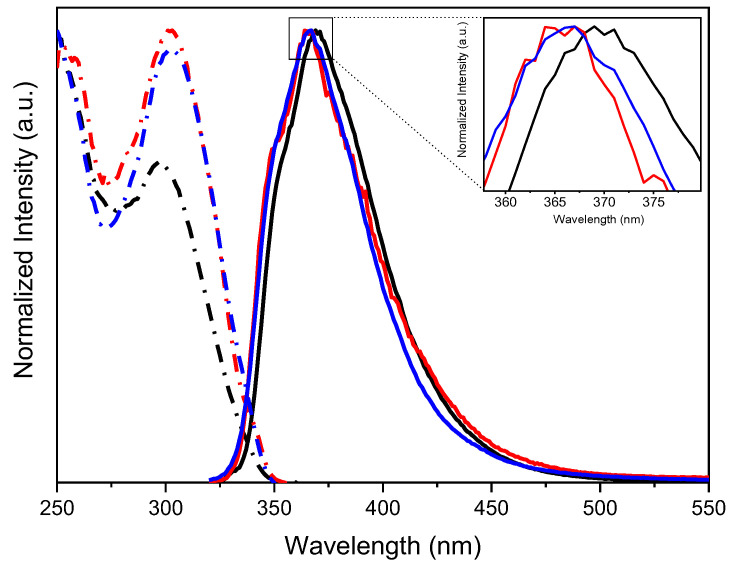
Normalized photoluminescence, PL, emission (continuous lines) and excitation (dashed–dotted lines) spectra of **TT-HThio**. DCM (1 × 10^−5^ M) at 298 K (black) and 77 K (red); λ_exc_ = 300 nm, λ_em_ = 370 nm. PMMA film (0.5% wt) at 298 K (blue); λ_exc_ = 300 nm, λ_em_ = 365 nm.

In diluted DCM solutions (1 × 10^−5^ M), **TT-HThio** displays a single fluorescence (FL) with photoluminescent quantum yield, Φ, equal to 11%; wavelength of the maximum peak (λ_em_) = 370 nm and lifetime (τ) = 0.81 ns at 298 K; and λ_em_ = 365 nm and τ =1.71 ns at 77 K (Figure 1, Figures S5 and S6). In PMMA-blended films (0.5 w% **TT-HThio**), one fluorescence (FL, Φ = 15%, λ_em_ = 365 nm, τ = 0.72 ns at 298 K, Figure 1 and Figure S19) is observed in exactly the same position as that measured in DCM at 77 K, as expected for a molecular emission in rigidified media.

**Figure 2:**
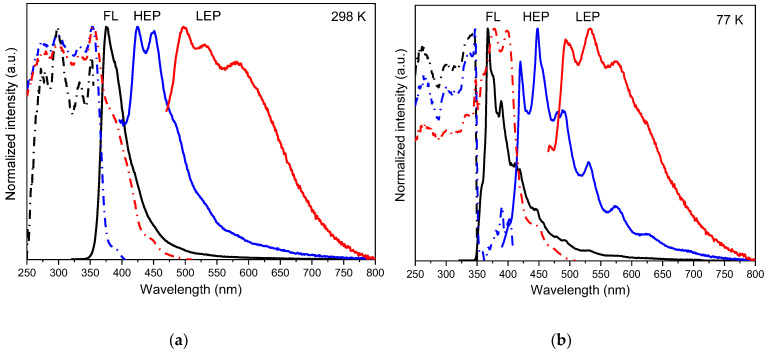
Normalized PL emission (continuous lines) and excitation (dashed–dotted lines) spectra of crystals of **TT-HThio**: (**a**) 298 K, emission at λ_exc_ = 300 nm (black), 376 nm (blue), 450 nm (red), excitation at λ_em_ = 376 nm (black), 426 nm (blue), 532 nm (red); (**b**) 77 K, emission at λ_exc_ = 300 nm (black), 368 nm (blue), 448 nm (red), excitation at λ_em_ = 368 nm (black), 420 nm (blue), 533 nm (red).

Crystals of **TT-HThio** show, both at 298 (Φ = 26%) and 77 K, an excitation-dependent PL behavior comprising one fluorescence and two phosphorescences (Figure 2). In particular, at 298 K, one fluorescence at λ_em_ = 376 nm (FL, τ = 1.15 ns, Figure S7) is observed by exciting at 300 nm, one high-energy phosphorescence (HEP, with vibronic replica at λ_em_ = 425 and 451 nm, τ = 5.91 ms, Figure S8) is visible by exciting at 376 nm and one low-energy phosphorescence (LEP, at λ_em_ = 497, 530 and 578 nm, τ = 50.22 ms, Figure S9) appears when exciting at 450 nm. At 77 K, the three components are better resolved vibronically, in almost the same positions but with longer lifetimes with respect to those measured at room temperature (FL, λ_em_ = 368 nm, τ = 1.48 ns; HEP, λ_em_ = 420 and 448 nm, τ = 10.61 ms; LEP, λ_em_ = 494, 533 and 577 nm, τ = 132.01 ms, Figures S10, S11 and S12, respectively). Intriguingly, the corresponding excitation profiles are characterized at low energy by three contributions recognizable at both 298 and 77 K. Specifically, one low-energy state (332, 354 nm at 298 K and 335, 347 at 77 K) assigned to singlet molecular excited state S_1_ in agreement with structural and theoretical studies (see later), one triplet excited state of molecular origin (389 nm at 298 K and 390, 403 nm at 77 K), T_mol_, and one triplet excited state of aggregate origin (446 nm at 298 K and 448, 471 nm at 77 K), T_ag_, are observed. A mirroring relationship with the corresponding emission is clearly visible at 77 K.

**Figure 3:**
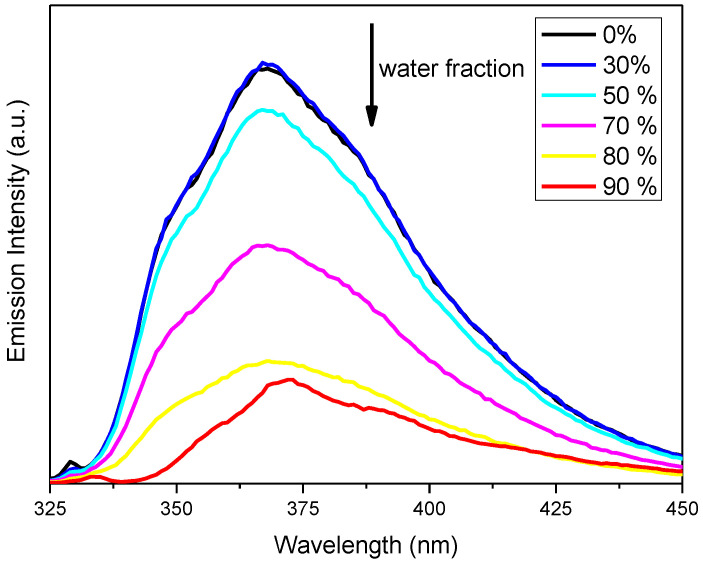
PL emission spectra of **TT-HThio** in THF with increasing water fractions; λ_exc_ = 300 nm.

Based on the observation that Φ increases from 11%, in diluted DCM solution, to 26%, measured for **TT-HThio** crystals, we hypothesized possible aggregation-enhanced emissive (AEE) features; therefore, solvent (THF)/non-solvent (water) experiments have been performed. Addition of increasing water fractions to **TT-HThio** in THF (keeping the concentration equal to 1 × 10^−5^ M) results, instead, in emission quenching (Figure 3), indicating that aggregation itself is not sufficient to intensify luminescence, and that proper molecule organization (i.e., crystallization) is an indispensable condition to enhance emission from **TT-HThio**. Similarly, in **TT** itself, crystallization is a necessary condition to switch on luminescence [9]. 

**Figure 4:**
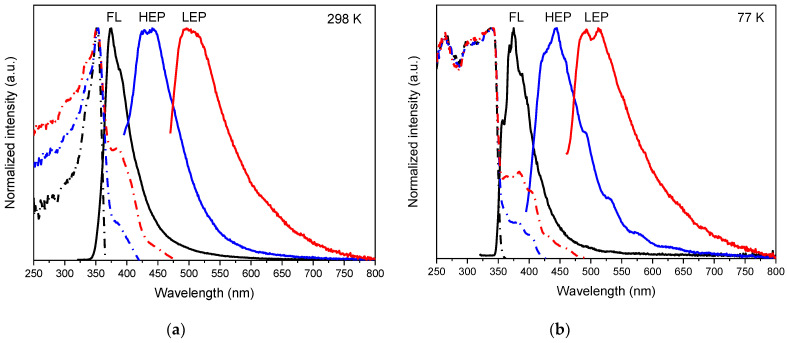
Normalized PL emission (continuous lines) and excitation (dashed–dotted lines) spectra of ground crystals of **TT-HThio**: (**a**) 298 K, emission at λ_exc_ = 300 nm (black), 376 nm (blue), 450 nm (red), excitation at λ_em_ = 365 nm (black), 434 nm (blue), 597 nm (red); (**b**) 77 K, emission at λ_exc_ = 300 nm (black), 376 nm (blue), 440 nm (red), excitation at λ_em_ = 375 nm (black), 443 nm (blue), 513 nm (red).

The relation between the photophysical properties of the sample and its crystallinity degree has been confirmed by a decrease in Φ (18%) and the loss of vibronic components when **TT-HThio** crystals are ground in a mortar, revealing mechanochromic behavior and confirming CEE features (Figure 4).

**Figure 5:**
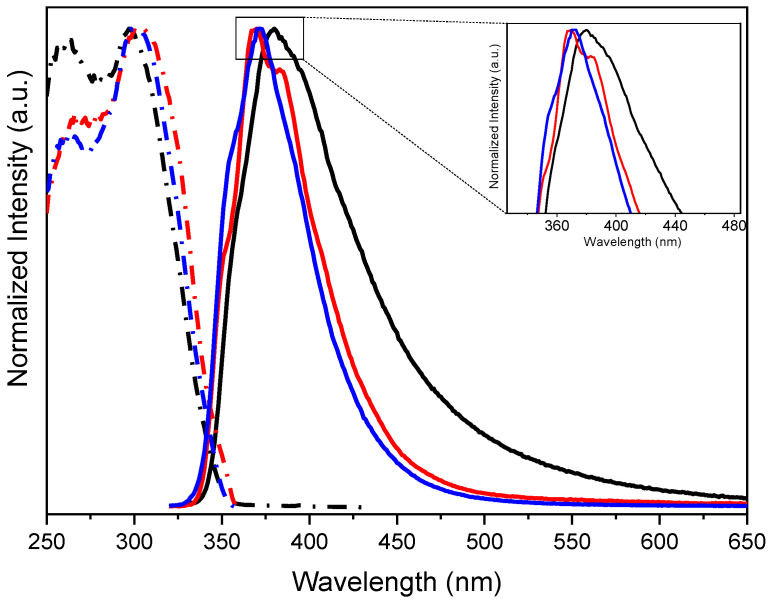
Normalized PL emission (continuous lines) and excitation (dashed–dotted lines) spectra of **TT-(HThio)_3_**. DCM (1 × 10^−5^ M) at 298 K (black) and 77 K (red); λ_exc_ = 300 nm, λ_em_ = 370 nm. PMMA film (0.5% wt) at 298 K (blue); λ_exc_ = 300 nm, λ_em_ = 370 nm.

In diluted DCM solutions (1 × 10^−5^ M), **TT-(HThio)_3_** displays a single fluorescence (FL, Φ = 5%, λ_em_ = 380 nm, τ = 1.17 ns at 298 K and λ_em_ = 370 nm, 1.42 ns at 77 K, Figure 5, Figures S20, S21). In PMMA-blended films (0.5 w% **TT-(HThio)_3_**), one fluorescence (FL, Φ = 17%, λ_em_ = 372 nm, τ = 0.90 ns at 298 K, Figure 5 and Figure S28) is observed in exactly the same position as that measured in DCM at 77 K. 

**Figure 6:**
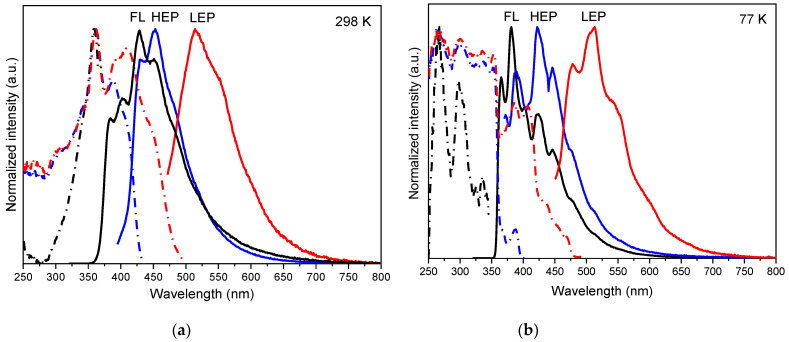
Normalized PL emission (continuous lines) and excitation (dashed–dotted lines) spectra of crystalline powders of **TT-(HThio)_3_**: (**a**) 298 K, emission at λ_exc_ = 300 nm (black), 384 nm (blue), 450 nm (red), excitation at λ_em_ = 382 nm (black), 452 nm (blue), 514 nm (red); (**b**) 77 K, emission at λ_exc_ = 300 nm (black), 360 nm (blue), 430 nm (red), excitation at λ_em_ = 365 nm (black), 423 nm (blue), 513 nm (red).

The solid-state photophysical characterization of **TT-(HThio)_3_** has been performed on crystalline powders (Φ = 22%), while different attempts to prepare single crystals suitable for XRD analysis failed. Similarly to **TT-HThio**, **TT-(HThio)_3_** displays at both 298 and 77 K an excitation-dependent PL behavior, comprising FL, HEP and LEP (Figure 6). In particular, at 298 K, FL at λ_em_ = 382, 400 nm (τ = 0.42 ns, Figure S22) is observed by exciting at 300 nm together with HEP at λ_em_ = 428, 453 nm (τ = 15.10 ms, Figure S23). The latter can be selectively activated by exciting at 384 nm. LEP at λ_em_ = 514, 550 nm (τ = 41.51 ms, Figure S24) becomes visible by exciting at sufficiently low energy (450 nm) to exclude the otherwise more intense FL and HEP. At 77 K, the three components are still present at λ_em_ = 364, 381 nm (FL, τ = 1.23 ns, Figure S25), λ_em_ = 423, 447 nm (HEP, τ = 153.42 ms, Figure S26) and λ_em_ = 492, 513 nm (LEP, τ = 327.16 ms, Figure S27). Again, in excitation profiles, one S_1_ state (360 nm at 298 K and 336, 354 at 77 K), one T_mol_ (387, 408 nm at 298 K and 386, 407 nm at 77 K), and one T_ag_ (448 nm at 298 K and 438, 468 nm at 77 K) are visible both at 298 and 77 K. The photophysical parameters of **TT-HThio** and **TT-(HThio)_3_** are summarized in Table 1.

**Figure 7:**
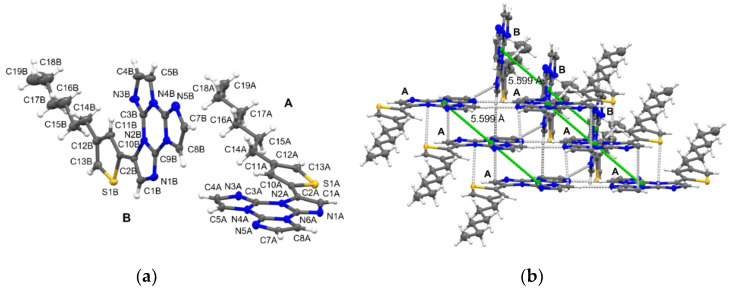

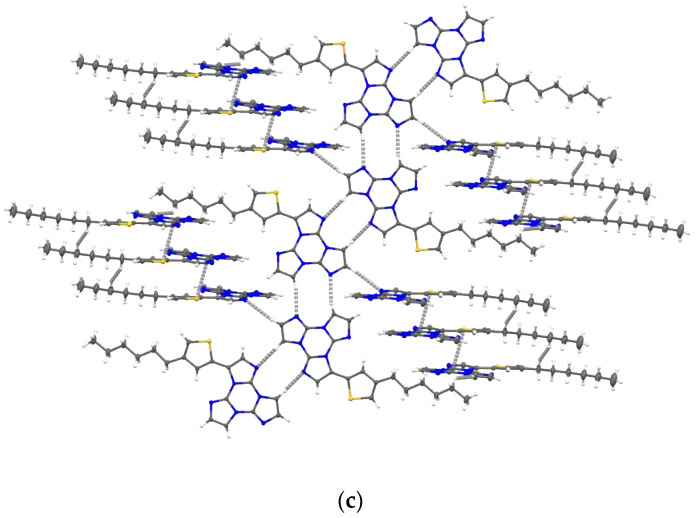
Ortep plots of **TT-HThio** showing: (**a**) the two molecules (A and B) of the asymmetric unit; (**b**) a fragment of crystal packing with shorter distances between triazinic geometrical centroids (green spheres) and C⋯C, C⋯S and C–H⋯π intermolecular contacts shorter than the sum of vdW radii (light grey–dashed lines); (**c**) the hydrogen bonded ribbon formed by A molecules and the π-stacks of B molecules. Ellipsoids at 20% probability.

Single crystals of **TT-HThio** suitable for X-ray diffraction analysis have been obtained by the slow evaporation of methanol solutions. **TT-HThio** crystallizes in the P-1 space group with two molecules in the asymmetric unit, A and B, forming very long colorless needles. In both A and B molecules, the TT and Thio moieties are almost coplanar, the least-squares planes through the two units are 14.12 and 6.00° in A and B, respectively (Figure 7a). The hexyl chain assumes in both cases an all-trans conformation. A and B molecules are almost orthogonal to each other (Figure 7b), both forming segregated head-to-head π-stacked aggregates with identical distance, equal to 5.599 Å, between the triazinic centroids of adjacent molecules. The shortest intermolecular contacts along the stacks are C1A⋅⋅⋅C8A_1+*x*,*y*,*z*_, 3.353(3), S1A⋅⋅⋅C9A_1+*x*,*y*,*z*_, 3.344(2) and C5A⋅⋅⋅C5A_1−*x*,1−*y*,1−*z*_, 3.376(3) Å (A stack) and S1B⋅⋅⋅C9B_1+*x*,*y*,*z*_, 3.388(2) Å (B stack). The TT moiety of molecule A is connected from both sides to other two centrosymmetry-related A molecules through quite short C–H⋅⋅⋅N hydrogen bonds (H5A⋅⋅⋅N5A_−*x*,1−*y*,1−*z*_, 2.38 Å; H8A⋅⋅⋅N1A_1−*x*,−*y*,1−*z*_, 2.43 Å), forming infinite ribbons from which the thio-hexyl chains depart (Figure 7c). The columnar aggregates of B molecules develop inside the hexyl chains of A molecules. A weak C–H⋅⋅⋅N hydrogen bond (H7A⋅⋅⋅N1B_−*x*,1−*y*,1−*z*_, 2.72 Å) connects A and B molecules with each other. 

## 3. Discussion

**Figure 8:**
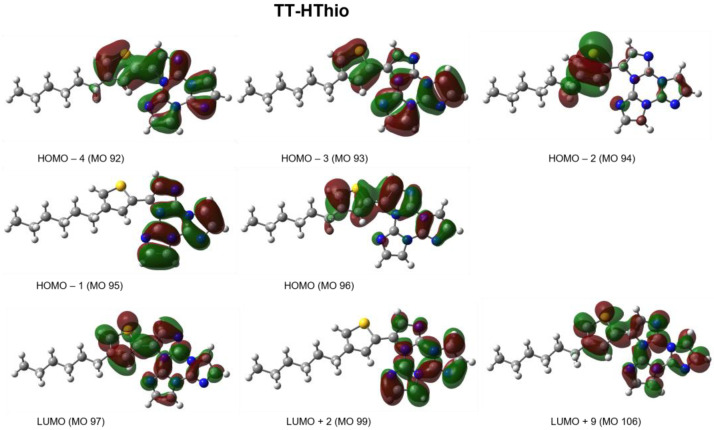
Plots of the ωB97X/6-311++G(d,p) MOs mainly involved in the lowest energy transitions of **TT-HThio** (isosurfaces value 0.02).

**Figure 9:**
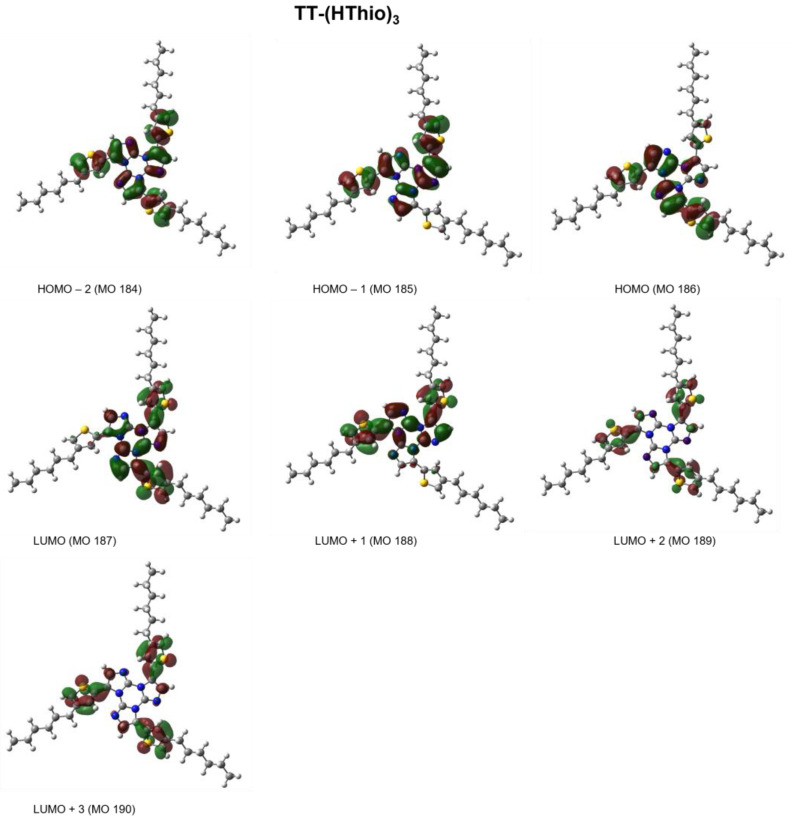
Plots of the ωB97X/6-311++G(d,p) MOs mainly involved in the lowest energy transitions of **TT-(HThio)_3_** (isosurfaces value 0.02).

As previously observed for a series of TT derivatives bearing one to three chromophoric substituents (i.e., 2-pyridine [25], fluoropyridine [24] or pyrene [26,27]) on the **TT** scaffold, the presence of the chromophore switches on fluorescence which is otherwise absent in **TT** itself in solution. From DFT/TDDFT calculations, it was inferred that **TT** lacks low-energy singlet states with non-null oscillator strength (*f*) due to the high symmetry of its electronic system. Analogously, “silent” low-energy singlet states are present in trihalogenated **TT** derivatives [21,23]. However, in monosubstituted **TT** derivatives, the disruption of the molecular symmetry results in S_1_ and upper levels with medium-to-high oscillator strength depending on the nature of the substituent itself. This is also noted for **TT** derivatives functionalized with three equal chromophoric fragments owing to the adopted non-planar conformation [27]. For **TT-HThio,** the S_1_ level is computed at 258 nm (*f* = 0.49), quite similar to the mono-pyridine **TT** derivative for which S_1_ is computed at 256 nm with *f* = 0.48. In both cases, S1 is mainly (88%) a HOMO→LUMO transition of (π,π*) type and very weak CT character (in the present case from thiophene to TT) (Figure 8 and Table S2). Moving to TT-(HThio)_3_ (Figure 9 and Table S3), it should be noted that the presence of three, rather than one, chromophoric substituents does not significantly affect the absorption and emission properties of the compound, as also observed for the mono-, di- and three-pyrene series of TT derivatives [27]. The absence of inter-chromophoric communication in multiple-substituted TT derivatives, evidenced also by electrochemical studies on **TT** and its halogenated derivatives [31], is associated with a splitting of the MO levels of the mono-derivative in two or three almost degenerate levels, originating excited S_n_ and T_n_ levels nearly isoenergetic with those of the mono-derivative (Table S3). For example, the first singlet levels of **TT-(HThio)_3_** are computed at 258 (S_1_, *f* = 0.005), 257 (S_2_, *f* = 0.86) and 257 nm (S_3_, *f* = 0.86), all of them of (π,π*) type with very weak CT character.

The observation of HEP in the solid state for both **TT-HThio** and **TT-(HThio)_3_** can be explained by examining the singlet–triplet energy gap, ΔE_ST_, separating S_1_ and the closer, lower-energy triplet state (T_n_) and the nature of this level. In the DFT freely optimized geometry of **TT-HThio**, starting from its X-ray molecular structure, a rather high (0.53 eV) energy gap is obtained. However, compared with S_1_, the closer triplet state, T_5_ (a 50% HOMO-2→LUMO and 20% HOMO-2→LUMO+9 (π,π*) transition) has a much more marked CT character, as deduced by looking at the involved HOMO-2 (Figure 8), which is essentially localized on thiophene only. The corresponding excited state dipoles, in fact, are 1.90 D (S_1_) and 3.37 D (T_5_). The different character of S_1_ and T_5_ suggests an easy intersystem crossing (ISC) which explains the HEP observed in crystals. Moreover, it should be noted that the optimized structure is characterized by much larger twisting than that observed in the X-ray structure. In fact, the C1-C2-C10-S1 torsion angle (ω, see Figure 7a for the atom numbering scheme) measures 38.3° in the optimized geometry, to be compared with the corresponding experimental torsions, 11.1(3)° and 7.4(3)° for molecules A and B, respectively, of the asymmetric unit. By fixing ω to the values assumed in the crystal structure, ΔE_ST_ decreases from 0.53 to 0.28 (ω = 11.1°) and 0.27 eV (ω = 7.4°), making the singlet-to-triplet ISC even easier. HEP is therefore associated with radiative decay from T_5_ itself or a lower triplet state T_mol_ after internal conversion (IC) from T_5_. It is visible in crystals thanks to the rigidifying effect and protection from oxygen quenching ascribable to intermolecular interactions. 

**Figure 10:**
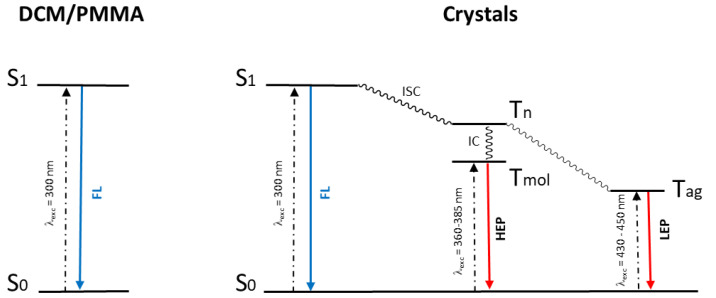
Energy level diagrams showing transitions associated with fluorescence (blue arrows) and phosphorescence (red arrows) for **TT-HThio** and **TT-HThio_3_** at 298 and 77 K.

The additional phosphorescent emission, LEP, can be associated with the presence of H-aggregates in the crystal structure, in agreement with previous findings on compounds with the same triazinic scaffold [9,10,21,22,23,24,25,26,27]. The schematic representation of the photophysical processes involved in the emissive behavior of both **TT-HThio** and **TT-(HThio)_3_** is reported in the Jablonski diagram below (Figure 10). It is interesting to compare the relative simplicity of this diagram with that, much more complicated, depicted for the analogous derivative with 2-pyridine, **TT-Py [25]**. Though the thiophene and pyridine chromophoric groups share rather similar electronic features, reflected for example in the similar emissive properties of their **TT** derivatives in DCM solution (Φ = 11%, λ_em_ = 370 nm for **TT-HThio**; Φ = 17%, λ_em_ = 351 nm for **TT-Py**), they are responsible for a different emissive behavior in their solid state. While crystals of both compounds display a high-energy phosphorescence of molecular origin (at 425, 451 nm for **TT-HThio** and at 408-418 nm, according to the crystalline phase, for **TT-Py**), associated with emission from a ^3^(π,π*) low-energy triplet level, **TT-Py** shows an additional long-lived molecular component, almost overlapped with the fluorescence. This latter emission was ascribed to radiative decay from a triplet state of mixed ^3^(σ/π,π*) symmetry, which is responsible for the observed dual anti-Kasha phosphorescence [32]. Such triplet states are absent in **TT-HThio**, whose frontier MOs have all π symmetry. Moreover, crystals of **TT-Py** display an additional low-energy fluorescence, associated with the higher mobility of the chromophoric pendant compared with the hexylthiophene group. 

In summary, two new members of the photophysically intriguing **TT** family have been isolated and characterized. The compounds, bearing one or three hexylthiophene fragments, display CEE behavior comprising fluorescence and heavy-atom-free dual phosphorescence, associated with molecular and supramolecular features. This work adds a new building block to the knowledge of this family and to RTP organic phenomena in general. From these results, the preparation of new multicomponent emitters can be envisaged. 

## 4. Materials and Methods

### 4.1. General Information

All reagents were purchased from chemical suppliers and used without further purification unless otherwise stated. **TTBr** and **TTBr_3_** were prepared according to published procedures [10,21]. Tributyl(4-hexylthiophen-2-yl)stannane was prepared according to a published procedure [33]. ^1^H and ^13^C NMR spectra were recorded on a Bruker AVANCE-400 instrument (400 MHz). Chemical shifts are reported in parts per million (ppm) and are referenced to the residual solvent peak (CH_2_Cl_2_, ^1^H: δ = 5.32 ppm, ^13^C: δ = 54.0 ppm); coupling constants (*J*) are given in hertz (Hz) and are quoted to the nearest 0.5 Hz. Peak multiplicities are described in the following way: s, singlet; d, doublet; t, triplet; p, pentet; m, multiplet. 

Films of **TT-HThio** and **TT-(HThio)_3_** dispersed in polymethylmethacrylate (PMMA) were prepared by spin coating (2000 rpm, 60 s) a dichloromethane solution (**TT-HThio** or **TT-(HThio)_3_**/PMMA = 0.5 wt %; PMMA = 10 wt % with respect to the solvent) on a quartz substrate.

### 4.2. Synthesis of **TT-HThio**

**TT-HThio** was prepared by Stille coupling between **TTBr** and tributyl(4-hexylthiophen-2-yl)stannane. In a typical reaction, **TTBr** (0.580 g; 2.10 mmol), tributyl(4-hexylthiophen-2-yl)stannane (0.959 g, 2.10 mmol), LiCl (1.000 g, 23.60 mmol), Pd(PPh_3_)_2_Cl_2_ (0.160 g, 0.210 mmol) and dry toluene (10 mL) are transferred inside a 100 mL dry Schlenk flask equipped with a magnetic stirrer. The mixture is de-aerated by means of three freeze–pump–thaw cycles. The system is heated under a static nitrogen atmosphere at 120 °C for 16 h. The reaction is then cooled to room temperature, filtered on Buchner and the solvent removed in vacuum. The crude reaction mixture is purified by flash chromatography on SiO_2_ with DCM/ACN as eluents (R_f_ = 0.53 in DCM/ACN = 8/2). The product is further purified by precipitation from DCM with hexane to give **TT-HThio** as a white solid (0.535 g, 1.47 mmol, Yield: 70%). Crystals suitable for X-ray diffraction analysis were obtained by slow evaporation from a MeOH solution.

*NMR data (9.4 T, CD_2_Cl_2_, 298 K, δ, ppm): ^1^H NMR* 7.80 (m, 2H), 7.53 (d, *J* = 1.2 Hz, 1H), 7.26 (m, 2H), 7.22 (d, *J* = 1.6 Hz, 1H), 7.07 (s, 1H), 2.69 (t, *J* = 7.1 Hz, 2H), 1.73 (p, *J* = 7.2 Hz, 2H), 1.46–1.30 (m, 6H), 0.95 (t, *J* = 6.9 Hz, 3H). *^13^C NMR* 144.3 (C), 136.4 (C), 136.0 (C), 135.8 (C), 132.3 (CH), 129.6 (CH), 129.1 (CH), 129.1 (CH), 128.1 (C), 123.1 (C), 122.3 (CH), 112.1 (CH), 111.7 (CH), 32.3 (CH_2_), 31.0 (CH_2_), 29.6 (CH_2_), 23.2 (CH_2_), 14.4 (CH_3_).

### 4.3. Synthesis of **TT-(HThio)_3_**

**TT-(HThio)_3_** was prepared by Stille coupling between **TTBr_3_** and tributyl(4-hexylthiophen-2-yl)stannane. In a typical reaction, **TTBr_3_** (0.400 g; 0.920 mmol), (tributyl(4-hexylthiophen-2-yl)stannane (1.264 g, 2.769 mmol), LiCl (0.390 g, 9.20 mmol), Pd(PPh_3_)_2_Cl_2_ (0.065 g, 0.092 mmol) and dry toluene (10 mL) are transferred inside a 100 mL dry Schlenk flask equipped with a magnetic stirrer. The mixture is de-aerated by means of three freeze–pump–thaw cycles. The system is heated under a static nitrogen atmosphere at 120 °C for 16 h. The reaction is then cooled to room temperature, filtered on Buchner and the solvent removed in vacuum. The crude reaction mixture is purified by flash chromatography on SiO_2_ with DCM/hexane as eluents (R_f_ = 0.72 in hexane/AcOEt = 8/2). The product is further purified by precipitation from DCM with MeOH to give **TT-(HThio)_3_** as a white solid (0.390 g, 0.56 mmol, Yield: 61%). Crystals suitable for X-ray diffraction analysis were obtained by slow evaporation from a MeOH solution.

*NMR data (9.4 T, CD_2_Cl_2_, 298 K, δ, ppm): ^1^H NMR NMR data (9.4 T, CD_2_Cl_2_, 298 K, d, ppm): ^1^H NMR* 7.43 (d, 1.10 Hz, 3H), 7.18 (s, 3H), 7.09 (d, 1.10 Hz, 3H), 2.68 (t, *J* = 7.5 Hz, 6H), 1.68 (p, *J* = 7.5 Hz, 6H), 1.48–1.30 (m, 18H), 0.90 (t, 6.9, 9H). *^13^C NMR* 143.9 (*C*), 136.4 (*C*), 132.3 (CH), 129.2 (CH), 128.0 (*C*),122.3 (CH), 122.3 (*C*), 32.1 (CH_2_), 30.8 (CH_2_), 29.40 (CH_2_), 23.0 (CH_2_), 14.3 (CH_3_). 

### 4.4. Single-Crystal X-ray Studies

X-ray data of **TT-HThio** were collected on a Bruker Apex II diffractometer (Bruker AXS Inc., Madison, WI, US) using MoKα radiation [34]. The structure was solved using direct methods and refined with SHELXL-14 [35] using a full-matrix least-squares procedure based on F^2^ using all data. Hydrogen atoms were placed at geometrically estimated positions. Details relating to the crystal and the structural refinement are presented in Table S1. Long bars of **TT-HThio** were grown at room temperature in a methanol solution of the compound. Full details of crystal data and structure refinement, in CIF format, are available as Supplementary Information. CCDC reference number: 1913040.

### 4.5. Computational Details

DFT and TDDFT calculations on **TT-HThio** and **TT-(HThio)_3_** were performed with the Gaussian 16 program (Revision A.03) [36] using the 6-311++G(d,p) basis set. The geometry of **TT-HThio** was optimized starting from the experimental molecular structure, as derived from X-ray studies. For comparison purposes, we adopted the same functional ωB97X [37] used for calculations of the previously reported parent cyclic triimidazole and its halogenated derivatives. 

### 4.6. Photophysical Characterization

Photoluminescence quantum yields were measured using a C11347 Quantaurus–Absolute Photoluminescence Quantum Yield Spectrometer (Hamamatsu Photonics K.K, Shizuoka, Japan) equipped with a 150 W Xenon lamp, an integrating sphere and a multichannel detector. Steady-state emission, excitation spectra and photoluminescence lifetimes were obtained using an FLS 980 (Edinburgh Instrument Ltd., Livingston, United Kingdom) spectrofluorimeter. The steady-state measurements were recorded with a 450 W Xenon arc lamp. Photoluminescence lifetime measurements were performed using an EPLED-300 (Edinburgh Instrument Ltd.) and microsecond flash Xe-lamp (60W, 0.1 ÷ 100 Hz) with the data acquisition devices time-correlated single-photon counting (TCSPC) and multi-channel scaling (MCS) methods, respectively. Average lifetimes were obtained as τ_av_ = $\frac{\sum A_{i}\tau_{i\;\;}^{2}}{\sum A_{i}\tau_{i}}$ from bi-exponential or three-exponential fits. Low-temperature measurements were performed by immersion of the sample in a liquid N_2_ quartz dewar.

## Figures and Tables

**Table 1:** Photophysical parameters of **TT-HThio** and **TT-(HThio)_3_**.

Compound			298 K			77 K	
			Φ (%)	λ_em_	τ_av_	λ_em_	τ_av_
**TT-HThio**	DCM		11	370	0.81 ns	365	1.71 ns
	PMMA		15	365	0.72 ns		
	Solid state	Crystals	26	376	1.15 ns	368	1.48 ns
				425, 451	5.91 ms	420, 448	10.61 ms
				497, 530, 578	50.22 ms	494, 533, 577	132.01 ms
		Ground Crystals	18	376	1.11 ns	375	1.91 ns
				428, 441	5.20 ms	423, 443	9.56 ms
				500	29.81 ms	492, 513	60.84 ms
**TT-(HThio)_3_**	DCM		5	380	1.17 ns	370	1.42 ns
	PMMA		17	372	0.90 ns		
	Crystalline powders		22	382, 400	0.42 ns	364, 381	1.23 ns
				428, 453	15.10 ms	423, 447	153.42 ms
				514, 550	41.51 ms	478, 512, 544	327.16 ms

## Data Availability

Not applicable.

## Supplementary Materials

The following supporting information can be downloaded at: https://www.mdpi.com/article/10.3390/molecules28010140/s1, Figures S1–S4: ^1^H and ^13^C NMR spectra; Table S1: crystal data; Figures S5–S30: photophysical data; Table S2–S3: computational data.

## Sample Availability

Samples of **TT-HThio** and **TT-(HThio)_3_** are available from the authors.

## References

[1] Qin W., Zhang P., Li H., Lam J.W.Y., Cai Y., Kwok R.T.K., Qian J., Zheng W., Tang B.Z. (2018). Ultrabright Red AIEgens for Two-Photon Vascular Imaging with High Resolution and Deep Penetration. Chem. Sci..

[2] Zhi J., Zhou Q., Shi H., An Z., Huang W. (2020). Organic Room Temperature Phosphorescence Materials for Biomedical Applications. Chem. Asian J..

[3] Gu L., Wu H., Ma H., Ye W., Jia W., Wang H., Chen H., Zhang N., Wang D., Qian C. (2020). Color-Tunable Ultralong Organic Room Temperature Phosphorescence from a Multicomponent Copolymer. Nat. Commun..

[4] Lei Y., Dai W., Guan J., Guo S., Ren F., Zhou Y., Shi J., Tong B., Cai Z., Zheng J. (2020). Wide-Range Color-Tunable Organic Phosphorescence Materials for Printable and Writable Security Inks. Angew. Chem. Int. Ed..

[5] Gao R., Yan D. (2017). Ordered Assembly of Hybrid Room-Temperature Phosphorescence Thin Films Showing Polarized Emission and the Sensing of VOCs. Chem. Commun..

[6] Hirata S., Totani K., Kaji H., Vacha M., Watanabe T., Adachi C. (2013). Reversible Thermal Recording Media Using Time-Dependent Persistent Room Temperature Phosphorescence. Adv. Opt. Mater..

[7] An Z., Zheng C., Tao Y., Chen R., Shi H., Chen T., Wang Z., Li H., Deng R., Liu X. (2015). Stabilizing Triplet Excited States for Ultralong Organic Phosphorescence. Nat. Mater..

[8] Gu L., Shi H., Bian L., Gu M., Ling K., Wang X., Ma H., Cai S., Ning W., Fu L. (2019). Colour-Tunable Ultra-Long Organic Phosphorescence of a Single-Component Molecular Crystal. Nat. Photonics.

[9] Lucenti E., Forni A., Botta C., Carlucci L., Giannini C., Marinotto D., Previtali A., Righetto S., Cariati E. (2017). H-Aggregates Granting Crystallization-Induced Emissive Behavior and Ultralong Phosphorescence from a Pure Organic Molecule. J. Phys. Chem. Lett..

[10] Lucenti E., Forni A., Botta C., Carlucci L., Giannini C., Marinotto D., Pavanello A., Previtali A., Righetto S., Cariati E. (2017). Cyclic Triimidazole Derivatives: Intriguing Examples of Multiple Emissions and Ultralong Phosphorescence at Room Temperature. Angew. Chem. Int. Ed..

[11] Mieno H., Kabe R., Notsuka N., Allendorf M.D., Adachi C. (2016). Long-Lived Room-Temperature Phosphorescence of Coronene in Zeolitic Imidazolate Framework ZIF-8. Adv. Opt. Mater..

[12] Kabe R., Adachi C. (2017). Organic Long Persistent Luminescence. Nature.

[13] Li D., Lu F., Wang J., Hu W., Cao X.M., Ma X., Tian H. (2018). Amorphous Metal-Free Room-Temperature Phosphorescent Small Molecules with Multicolor Photoluminescence via a Host-Guest and Dual-Emission Strategy. J. Am. Chem. Soc..

[14] Zhang Z.Y., Xu W.W., Xu W.S., Niu J., Sun X.H., Liu Y. (2020). A Synergistic Enhancement Strategy for Realizing Ultralong and Efficient Room-Temperature Phosphorescence. Angew. Chem. Int. Ed..

[15] Hayduk M., Riebe S., Voskuhl J. (2018). Phosphorescence through Hindered Motion of Pure Organic Emitters. Chem. Eur. J..

[16] Baroncini M., Bergamini G., Ceroni P. (2017). Rigidification or Interaction-Induced Phosphorescence of Organic Molecules. Chem. Commun..

[17] Sun L., Zhu W., Yang F., Li B., Ren X., Zhang X., Hu W. (2018). Molecular Cocrystals: Design, Charge-Transfer and Optoelectronic Functionality. Phys. Chem. Chem. Phys..

[18] Bolton O., Lee K., Kim H.-J., Lin K.Y., Kim J. (2011). Activating Efficient Phosphorescence from Purely Organic Materials by Crystal Design. Nat. Chem..

[19] Shi H., An Z., Li P.-Z., Yin J., Xing G., He T., Chen H., Wang J., Sun H., Huang W. (2016). Enhancing Organic Phosphorescence by Manipulating Heavy-Atom Interaction. Cryst. Growth Des..

[20] Lin Z., Kabe R., Nishimura N., Jinnai K., Adachi C. (2018). Organic Long-Persistent Luminescence from a Flexible and Transparent Doped Polymer. Adv. Mater..

[21] Lucenti E., Forni A., Botta C., Carlucci L., Colombo A., Giannini C., Marinotto D., Previtali A., Righetto S., Cariati E. (2018). The Effect of Bromo Substituents on the Multifaceted Emissive and Crystal-Packing Features of Cyclic Triimidazole Derivatives. ChemPhotoChem.

[22] Lucenti E., Forni A., Botta C., Giannini C., Malpicci D., Marinotto D., Previtali A., Righetto S., Cariati E. (2019). Intrinsic and Extrinsic Heavy-Atom Effects on the Multifaceted Emissive Behavior of Cyclic Triimidazole. Chem. Eur. J..

[23] Giannini C., Forni A., Malpicci D., Lucenti E., Marinotto D., Previtali A., Carlucci L., Cariati E. (2021). Room Temperature Phosphorescence from Organic Materials: Unravelling the Emissive Behaviour of Chloro-Substituted Derivatives of Cyclic Triimidazole. Eur. J. Org. Chem..

[24] Previtali A., Lucenti E., Forni A., Mauri L., Botta C., Giannini C., Malpicci D., Marinotto D., Righetto S., Cariati E. (2019). Solid State Room Temperature Dual Phosphorescence from 3-(2-Fluoropyridin-4-Yl)Triimidazo [1,2-*a*:1′,2′-*c*:1″,2″-*e*][1,3,5]Triazine. Molecules.

[25] Lucenti E., Forni A., Previtali A., Marinotto D., Malpicci D., Righetto S., Giannini C., Virgili T., Kabacinski P., Ganzer L. (2020). Unravelling the Intricate Photophysical Behavior of 3-(Pyridin-2-Yl)Triimidazotriazine AIE and RTP Polymorphs. Chem. Sci..

[26] Previtali A., He W., Forni A., Malpicci D., Lucenti E., Marinotto D., Carlucci L., Mercandelli P., Ortenzi M.A., Terraneo G. (2021). Tunable Linear and Nonlinear Optical Properties from Room Temperature Phosphorescent Cyclic Triimidazole-Pyrene Bio-Probe. Chem. Eur. J..

[27] Malpicci D., Giannini C., Lucenti E., Forni A., Marinotto D., Cariati E. (2021). Mono-, Di-, Tri-Pyrene Substituted Cyclic Triimidazole: A Family of Highly Emissive and RTP Chromophores. Photochem.

[28] Formenti M., Blasi D., Cariati E., Carlucci L., Forni A., Giannini C., Guidotti M., Econdi S., Malpicci D., Marinotto D. (2022). Pyrene-Substituted Cyclic Triimidazole: An Appealing and Versatile Luminescent Scaffold for Explosive Detection. Dye Pigment.

[29] Ikeya M., Katada G., Ito S. (2019). Tunable Mechanochromic Luminescence of 2-Alkyl-4-(Pyren-1-Yl)Thiophenes: Controlling the Self-Recovering Properties and the Range of Chromism. Chem. Commun..

[30] Yoshida R., Tachikawa T., Ito S. (2022). Mechano- and Thermo-Responsive Luminescence of Crystalline Thienylbenzothiadiazole Derivatives: Stepwise Hypsochromic Switching of Near-Infrared Emission. Cryst. Growth Des..

[31] Magni M., Lucenti E., Previtali A., Mussini P.R., Cariati E. (2019). Electrochemistry of Cyclic Triimidazoles and Their Halo Derivatives: A Casebook for Multiple Equivalent Centers and Electrocatalysis. Electrochim. Acta.

[32] Malpicci D., Lucenti E., Giannini C., Forni A., Botta C., Cariati E. (2021). Prompt and Long-Lived Anti-Kasha Emission from Organic Dyes. Molecules.

[33] Kuo C.Y., Huang Y.C., Hsiow C.Y., Yang Y.W., Huang C.I., Rwei S.P., Wang H.L., Wang L. (2013). Effect of Side-Chain Architecture on the Optical and Crystalline Properties of Two-Dimensional Polythiophenes. Macromolecules.

[34] Bruker (1997). SMART, SAINT and SADABS.

[35] Sheldrick G.M. (2015). Crystal Structure Refinement with SHELXL. Acta Cryst. Sect. C Struct. Chem..

[36] Frisch M.J., Trucks G.W., Schlegel H.B., Scuseria G.E., Robb M.A., Cheeseman J.R., Scalmani G., Barone V., Petersson G.A., Nakatsuji H. (2016). Gaussian 16.

[37] Chai J.-D., Head-Gordon M. (2008). Systematic Optimization of Long-Range Corrected Hybrid Density Functionals. J. Chem. Phys..

