# Supplementary material for: Crystallization-Enhanced Emission and Room-Temperature Phosphorescence of Cyclic Triimidazole-Monohexyl Thiophene Derivatives
Source: Molecules. 2022 Dec 24;28(1):140. doi: 10.3390/molecules28010140 (PMC9822294; doi:10.3390/molecules28010140)
Supplement: Supplementary file 1 [file molecules-28-00140-s001.zip › Supplementary Information.pdf]

Crystallization Enhanced Emission and Room Temperature Phosphorescence of cyclic triimidazole-monohexyl thiophene derivatives

Daniele Malpicci [1,2], Alessandra Forni [2,3*], Elena Cariati [1,2,3], Riku Inoguchi [4], Daniele Marinotto [2,3], Daniele Maver [1,2], Federico Turco [1] and Elena Lucenti [2,3*]

Supplementary information

Table of Contents

1. NMR Spectra 2
2. Crystal data 8
3. Photophysical Data 9
4. AIE Tests 21
5. Theoretical Studies 22

1. NMR Spectra

Figure S1. ^{1}H-NMR spectra (CD_2Cl_2, 400 MHz) of **TT-HThio** (top) with expansion of aromatic and aliphatic region (below)

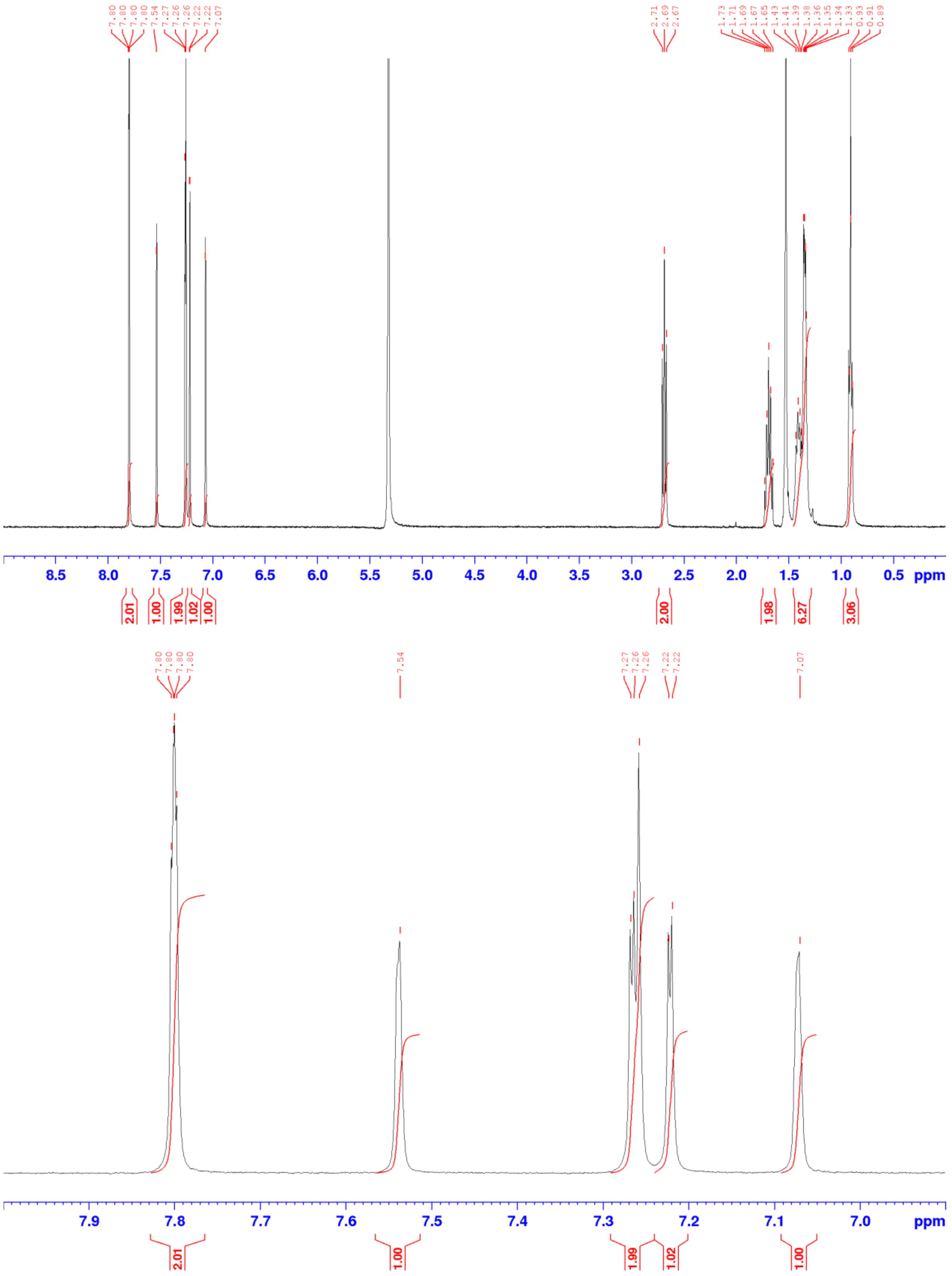

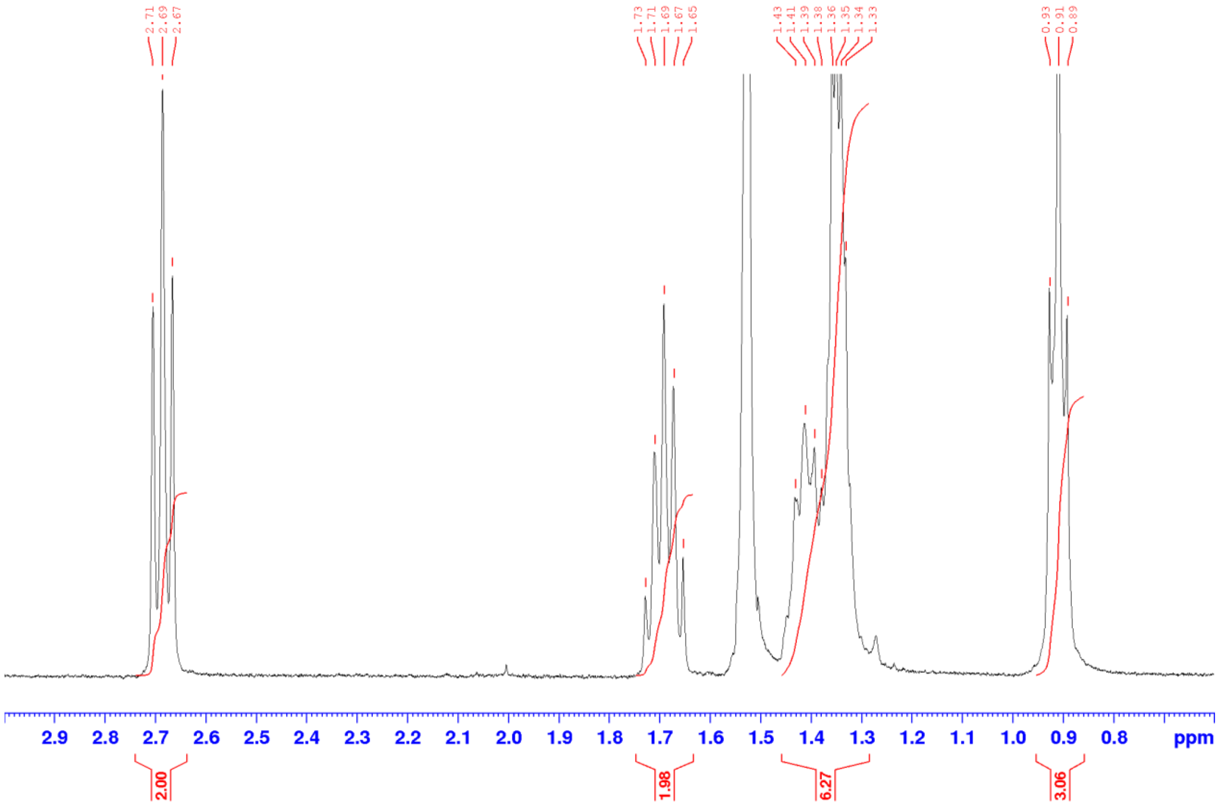

Figure S2. ^{13}C-NMR spectra (CD_2Cl_2, 100 MHz) of **TT-HThio** (top) with expansion of aromatic and aliphatic region (below)

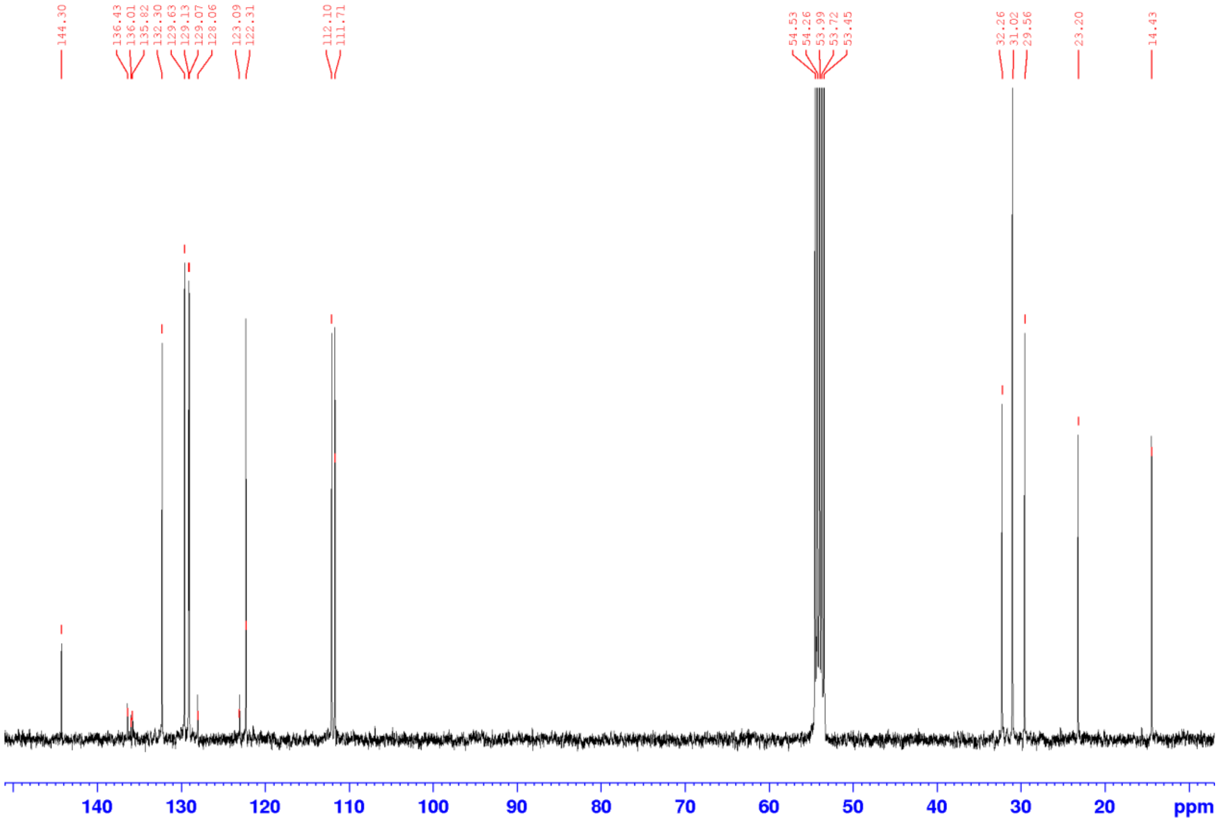

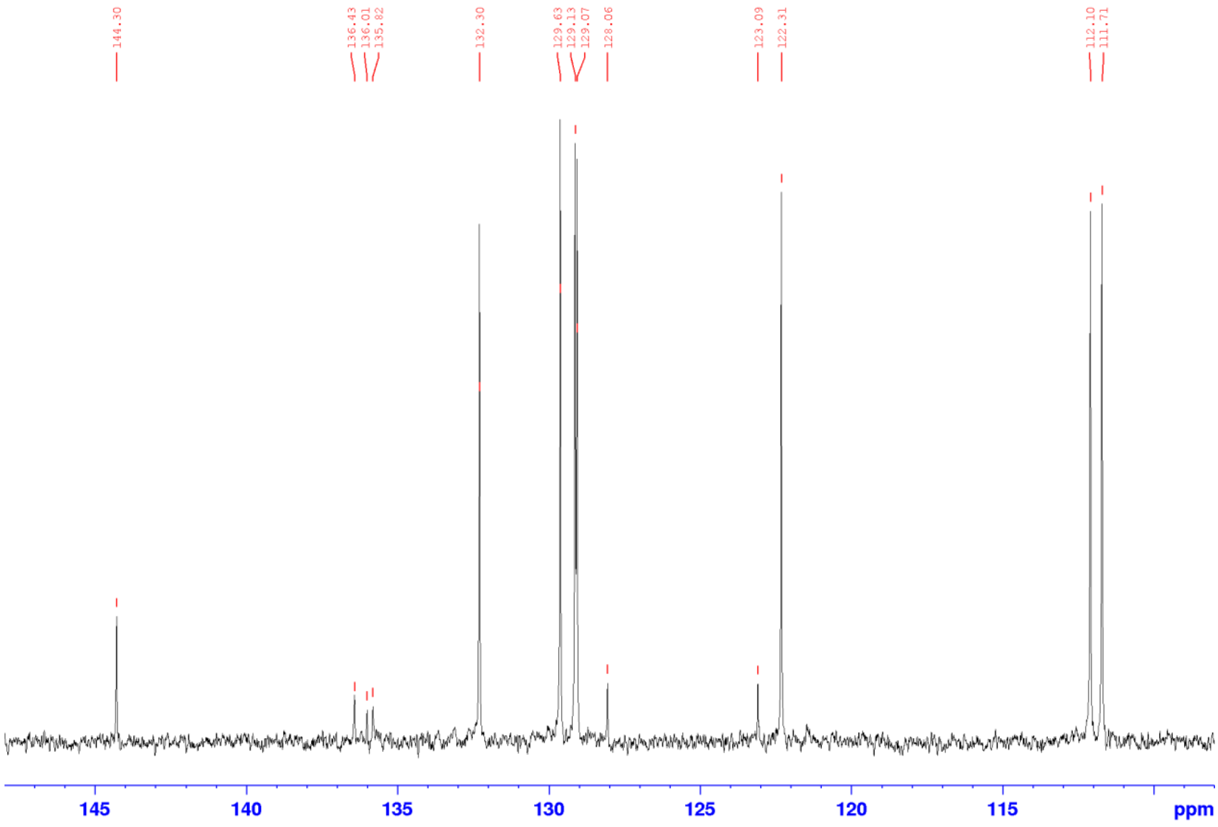
144.30
136.43
136.01
135.82
132.30
129.63
129.13
129.07
128.06
123.09
122.31
112.10
111.71
145
140
135
130
125
120
115
ppm

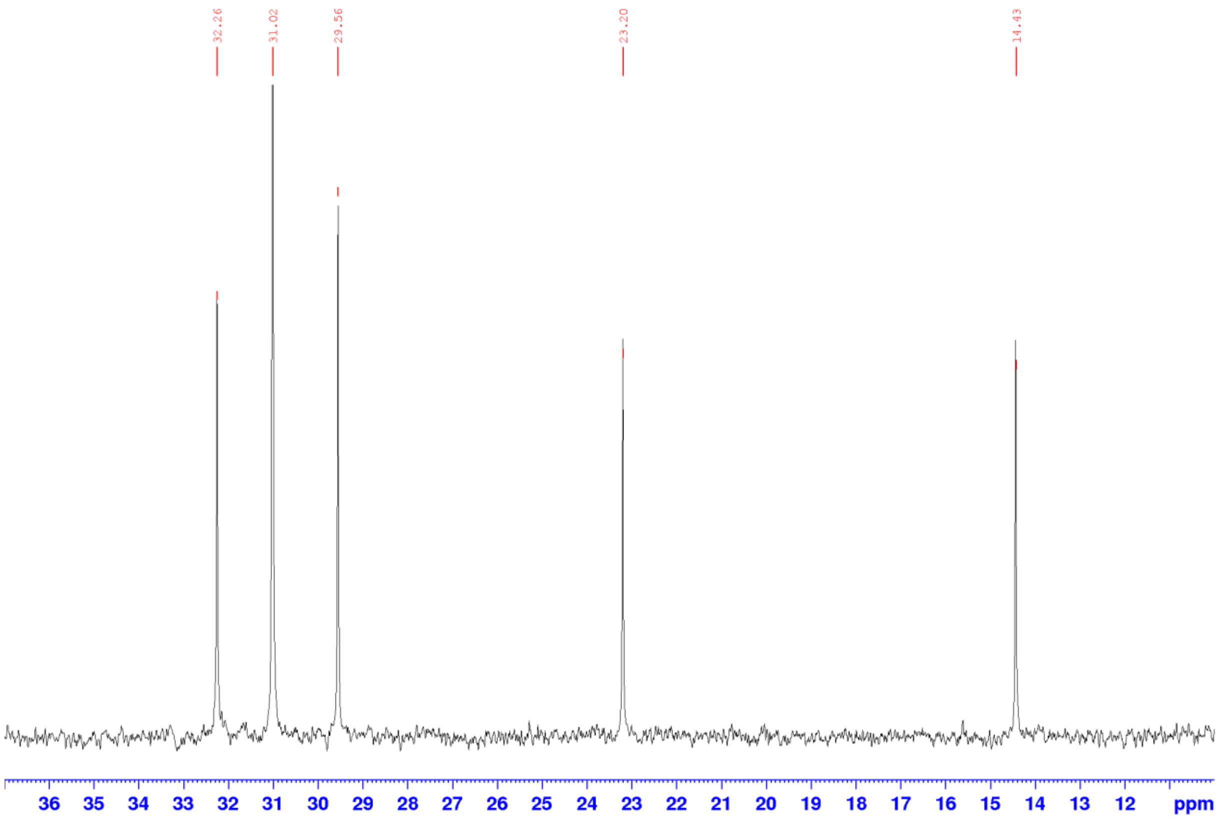
32.26
31.02
29.56
23.20
14.43
36
35
34
33
32
31
30
29
28
27
26
25
24
23
22
21
20
19
18
17
16
15
14
13
12
ppm

Figure S3. ^{1}H-NMR spectra (CD_2Cl_2, 400 MHz) of **TT-(HThio)$_3$** (top) with expansion of aromatic and aliphatic region (below)

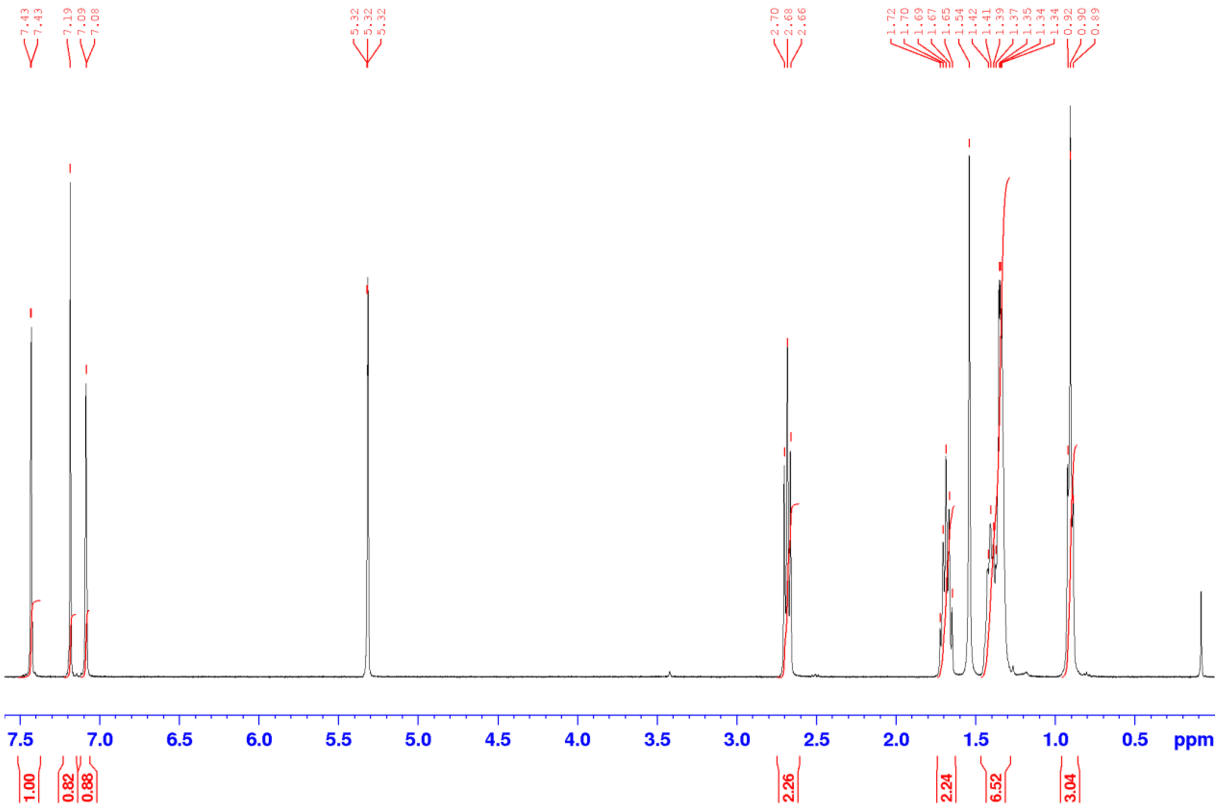

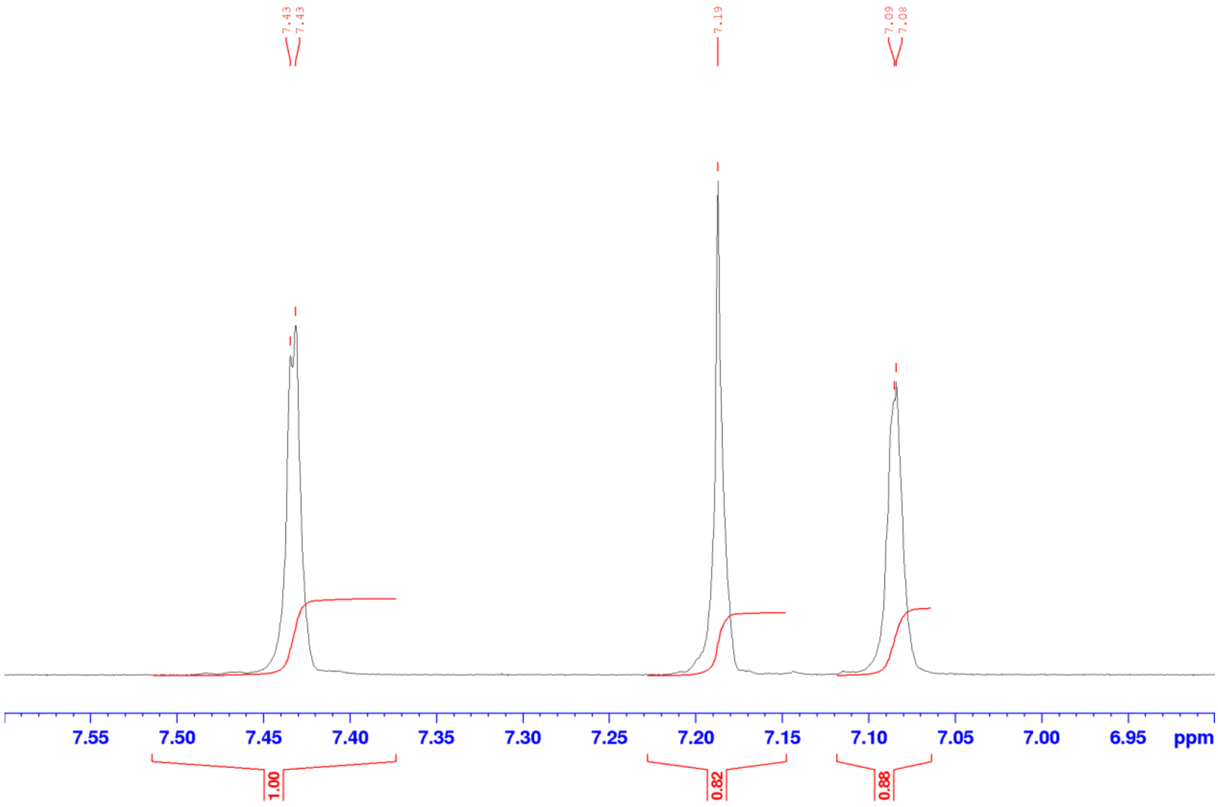

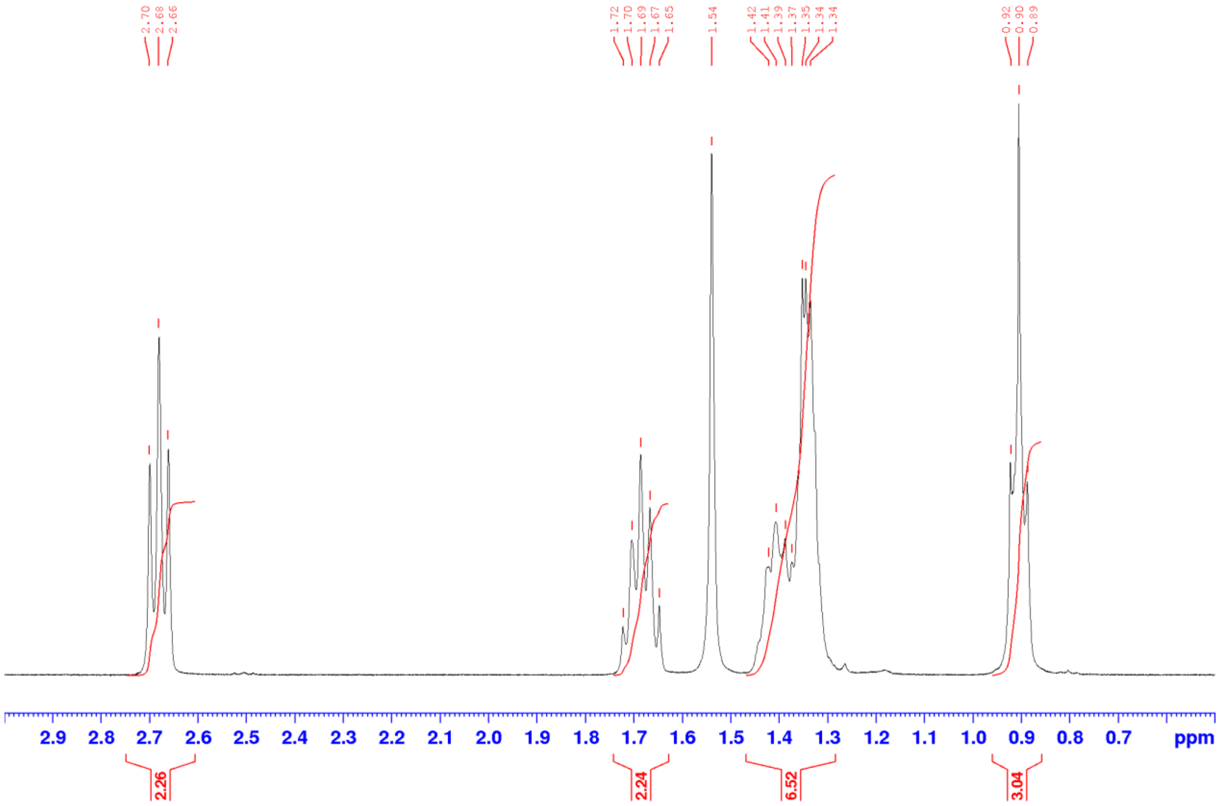

Figure S4. ^{13}C-NMR spectra (CD_2Cl_2, 100 MHz) of **TT-(HThio)$_3$** (top) with expansion of aromatic and aliphatic region (below)

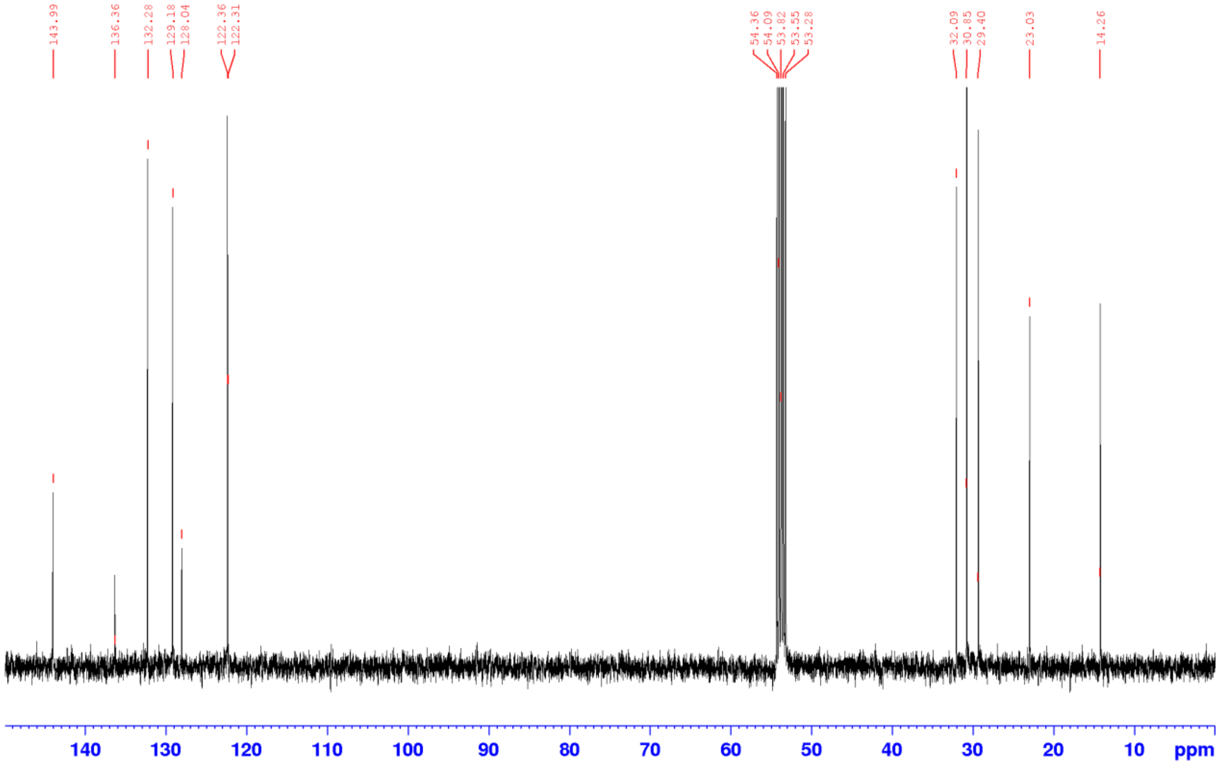

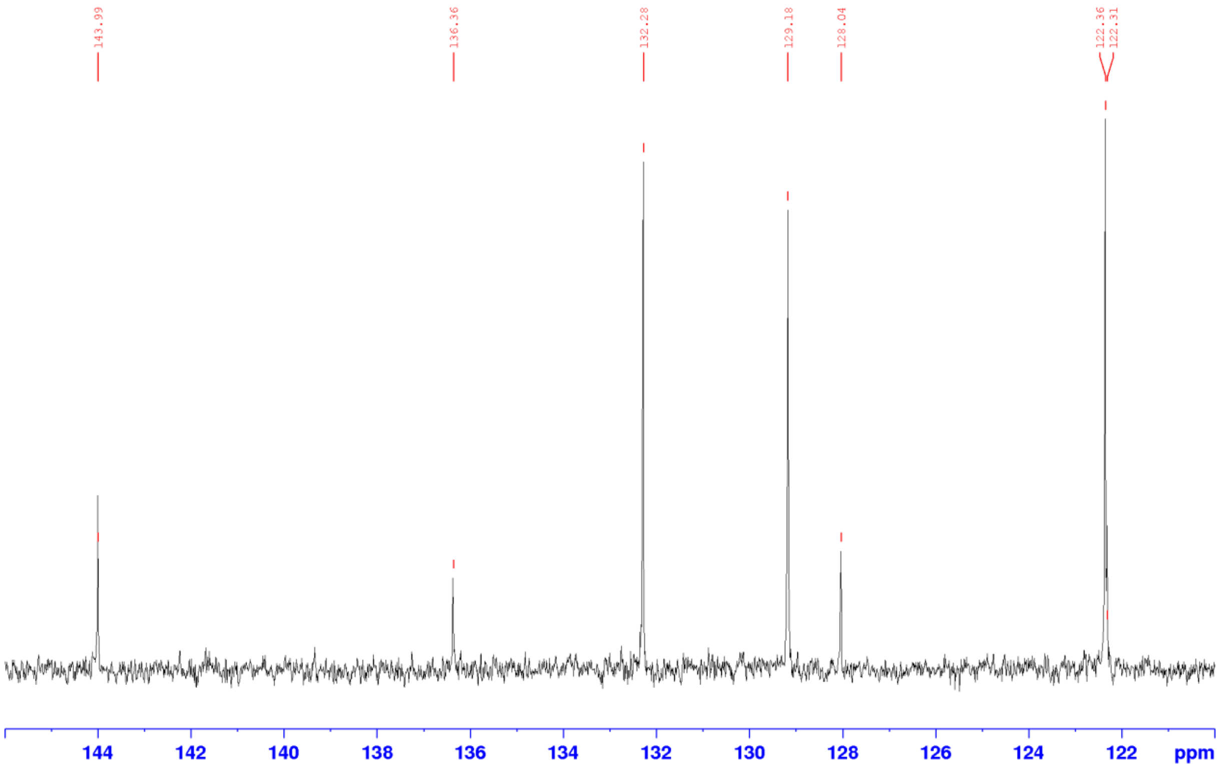
143.99
136.36
132.28
129.18
128.04
122.36
122.31
144
142
140
138
136
134
132
130
128
126
124
122
ppm

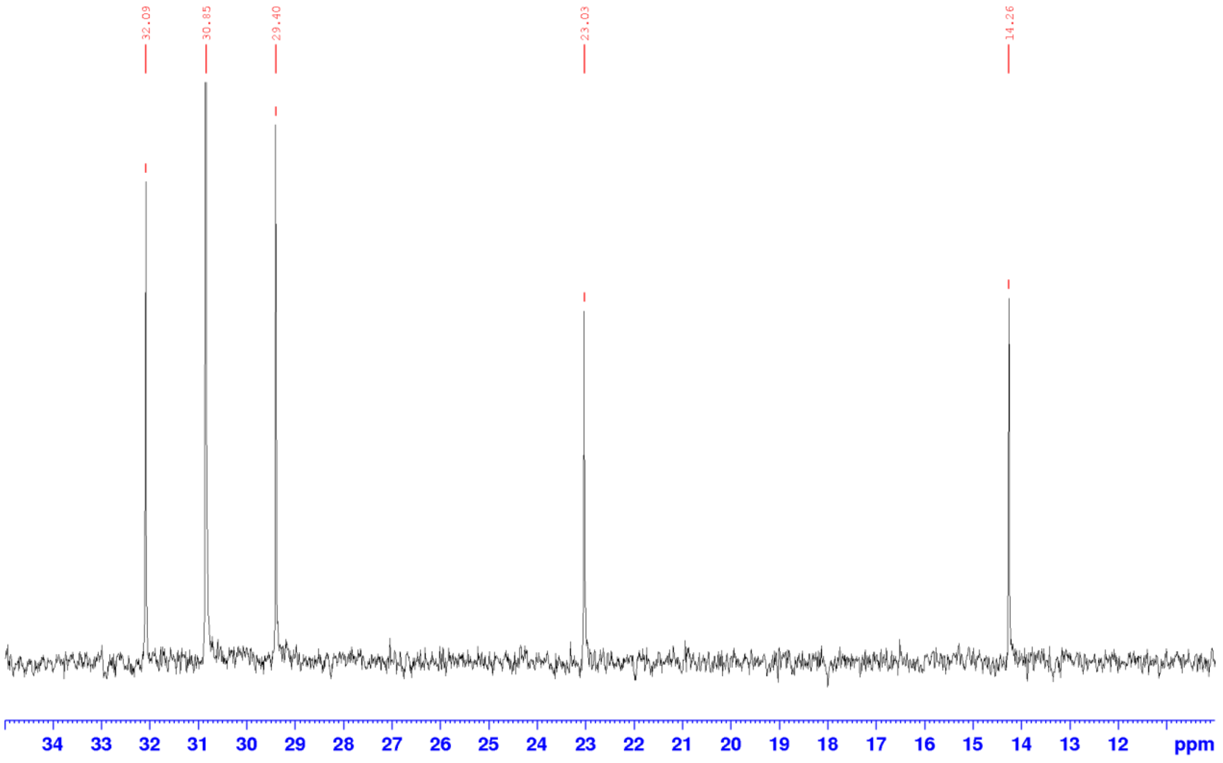
32.09
30.85
29.40
23.03
14.26
34
33
32
31
30
29
28
27
26
25
24
23
22
21
20
19
18
17
16
15
14
13
12
ppm

2. Crystal data

Table S1 Crystal data, data collection and refinement details for **TT-HThio**

	**TT-HThio**
Crystal data	
Chemical formula	$C_{19}H_{20}N_6S$
M_r	364.47
Crystal system	triclinic
Space group	P-1 (No. 2)
Temperature [K]	294(2)
a [Å]	5.5991(5)
b [Å]	13.0155(12)
c [Å]	24.977(2)
α [°]	85.150(2)
β [°]	88.236(2)
γ [°]	81.751(2)
V [Å^3]	1794.6(3)
Z	4
μ(MoKα) [mm^{-1}]	0.196
Crystal size [mm]	0.52 × 0.12 × 0.03
Data collection	
No. of measured reflections	36009
No. of independent reflections	10883
No. of observed reflections [I > 2σ(I)]	6936
R_{int}	0.042
$R_σ$	0.045
$(\sin θ/λ)_{max}$ [Å^{-1}]	0.708
Refinement	
$R[F^2 > 2σ(F^2)]$	0.0642
$wR(F^2)$	0.1534
S	1.020
No. of reflections	10883
No. of parameters	471
No. of restraints	0
$Δρ_{max}$, $Δρ_{min}$ (e Å^{-3})	0.357, –0.264

3. Photophysical Data

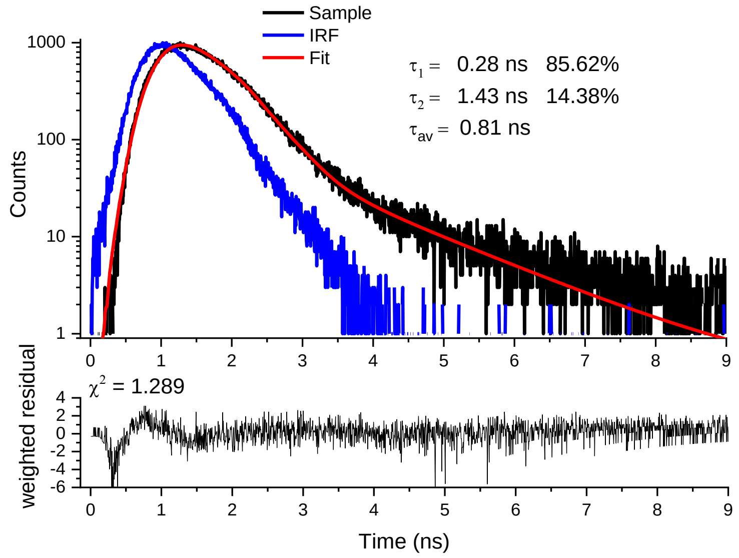

Figure S5. Lifetime measurement (λ_{exc} = 300 nm, λ_{em} = 369 nm) of **TT-HThio** in DCM $1{\cdot}10^{-5}$ M at 298 K.

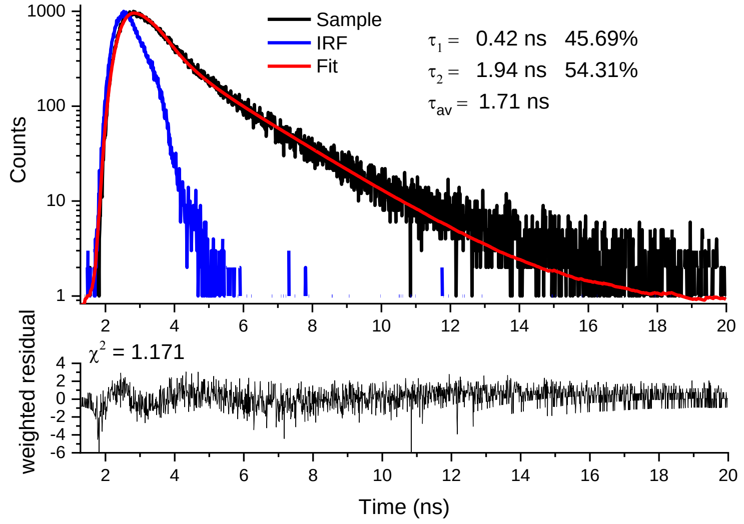

Figure S6. Lifetime measurement (λ_{exc} = 300 nm, λ_{em} = 366 nm) of **TT-HThio** in DCM $1{\cdot}10^{-5}$ M at 77 K.

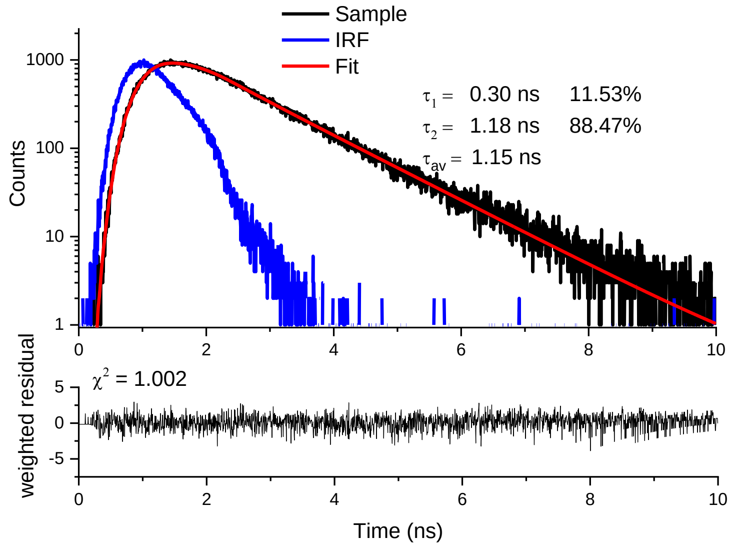

Figure S7. Lifetime measurement (λ_{exc} = 300 nm, λ_{em} = 376 nm) of **TT-HThio** crystals at 298 K.

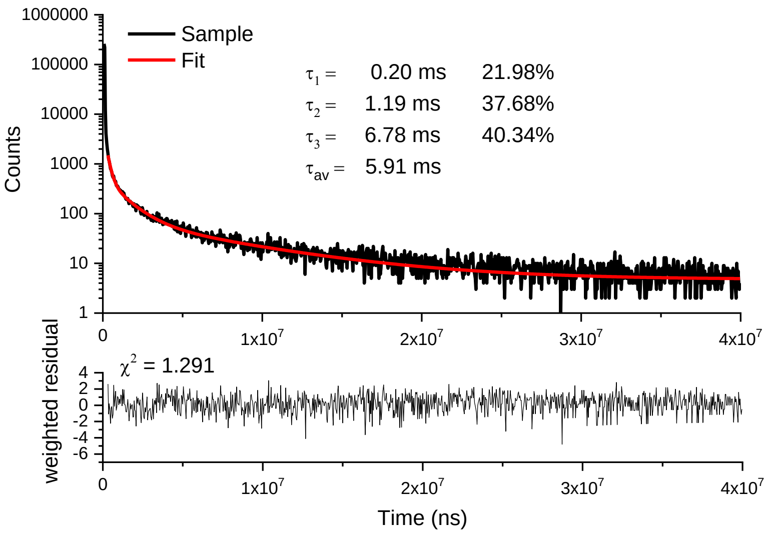

Figure S8. Lifetime measurement (λ_{exc} = 300 nm, λ_{em} = 425 nm) of **TT-HThio** crystals at 298 K.

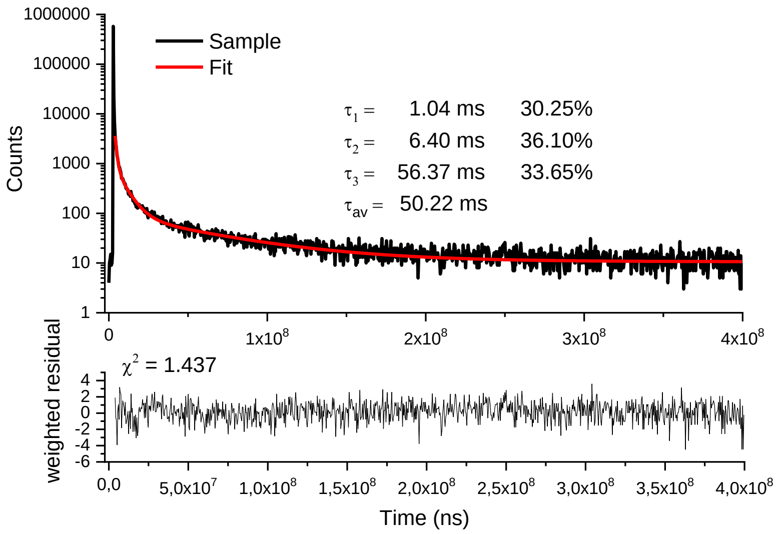

Figure S9. Lifetime measurement (λ_{exc} = 300 nm, λ_{em} = 532 nm) of **TT-HThio** crystals at 298 K.

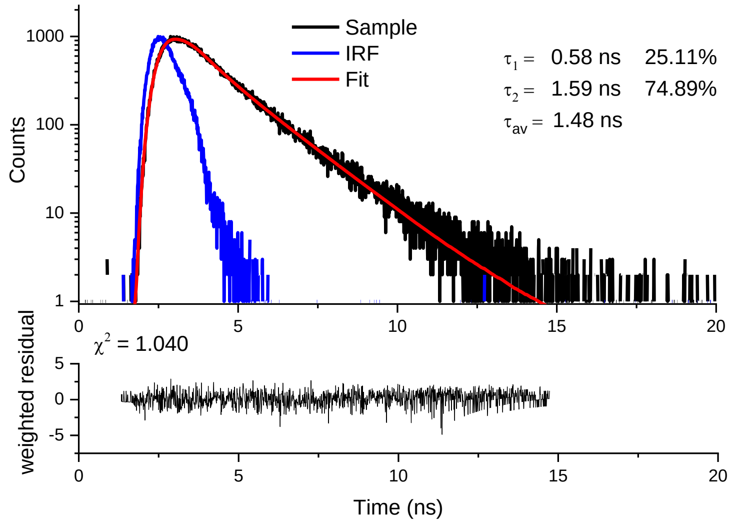

Figure S10. Lifetime measurement (λ_{exc} = 300 nm, λ_{em} = 368 nm) of **TT-HThio** crystals at 77 K.

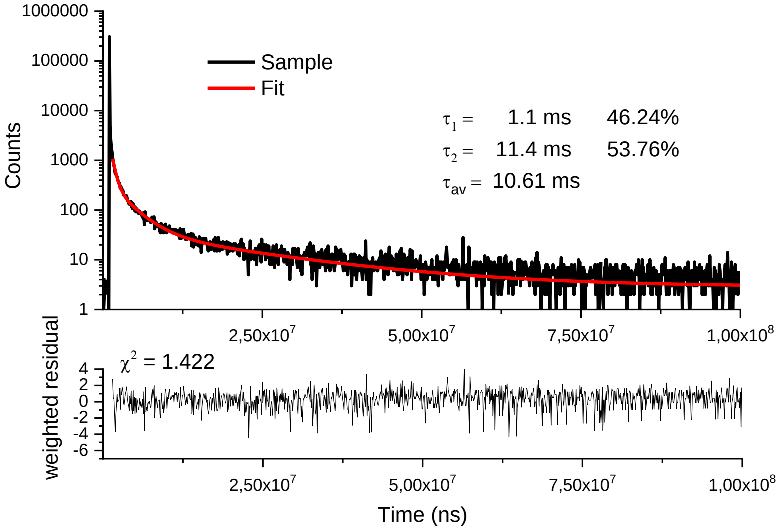

Figure S11. Lifetime measurement (λ_{exc} = 300 nm, λ_{em} = 420 nm) of **TT-HThio** crystals at 77 K.

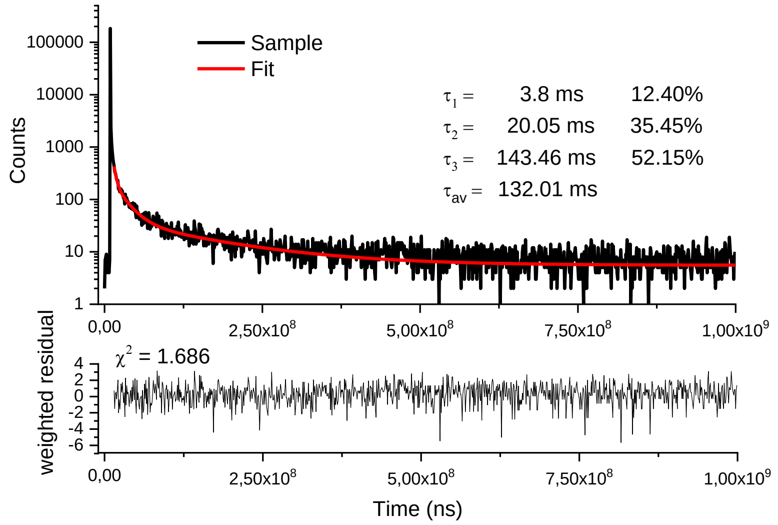

Figure S12. Lifetime measurement (λ_{exc} = 300 nm, λ_{em} = 533 nm) of **TT-HThio** crystals at 77 K.

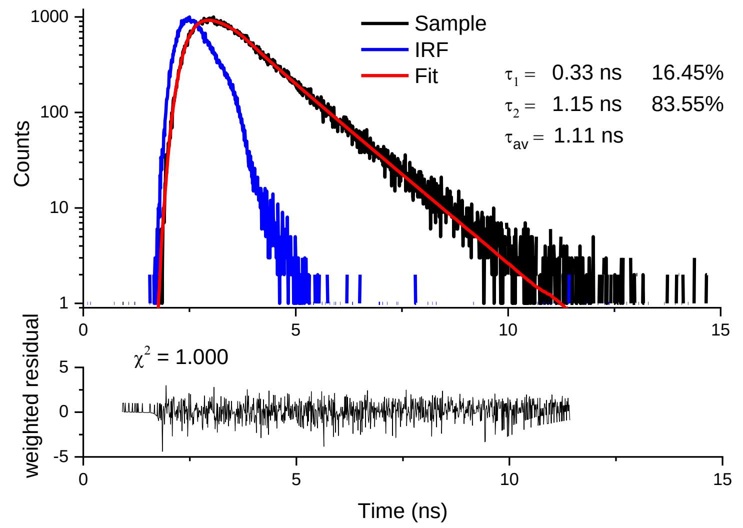

Figure S13. Lifetime measurement (λ_{exc} = 300 nm, λ_{em} = 375 nm) of **TT-HThio** ground crystals at 298 K.

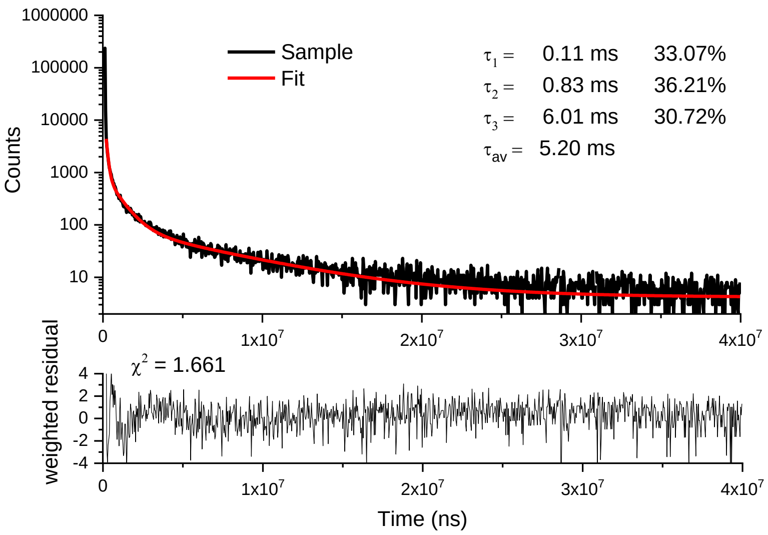

Figure S14. Lifetime measurement (λ_{exc} = 300 nm, λ_{em} = 434 nm) of **TT-HThio** ground crystals at 298 K.

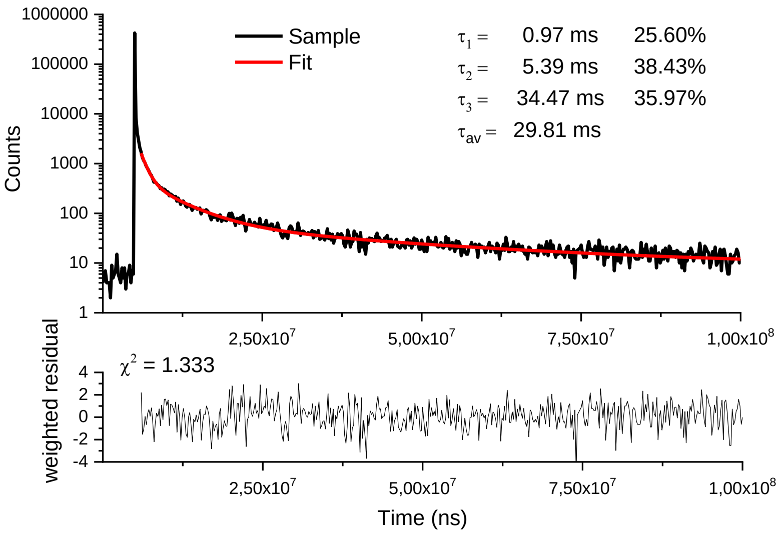

Figure S15. Lifetime measurement (λ_{exc} = 300 nm, λ_{em} = 530 nm) of **TT-HThio** ground crystals at 298 K.

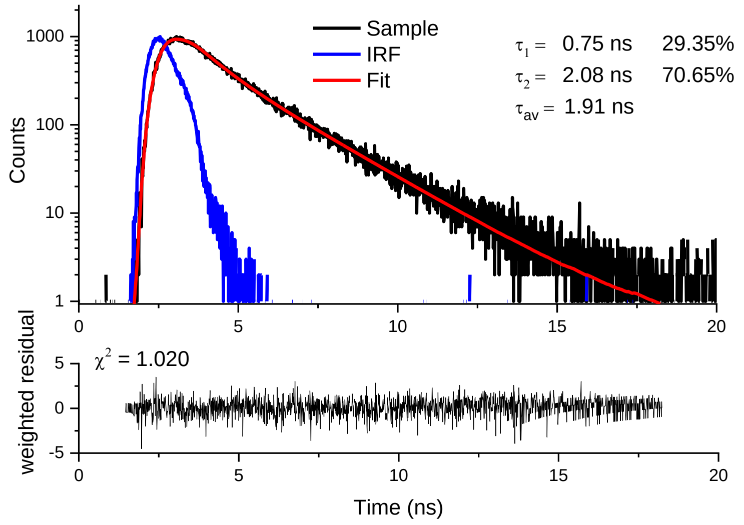

Figure S16. Lifetime measurement (λ_{exc} = 300 nm, λ_{em} = 375 nm) of **TT-HThio** ground crystals at 77 K.

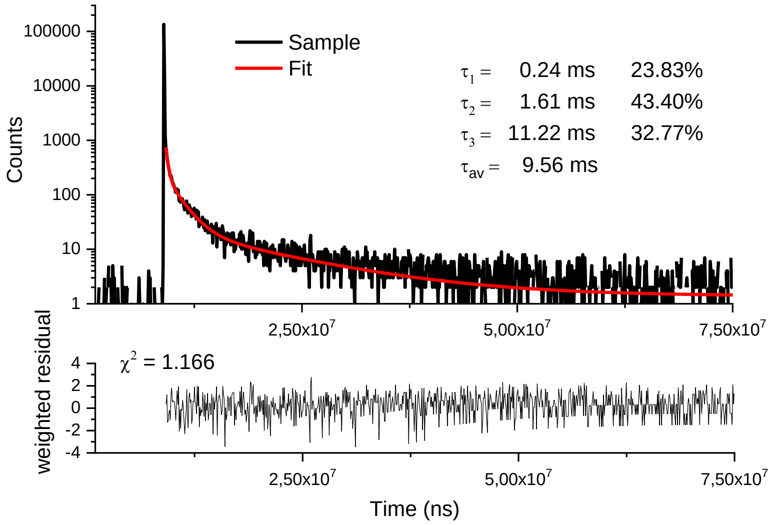

Figure S17. Lifetime measurement (λ_{exc} = 300 nm, λ_{em} = 430 nm) of **TT-HThio** ground crystals at 77 K.

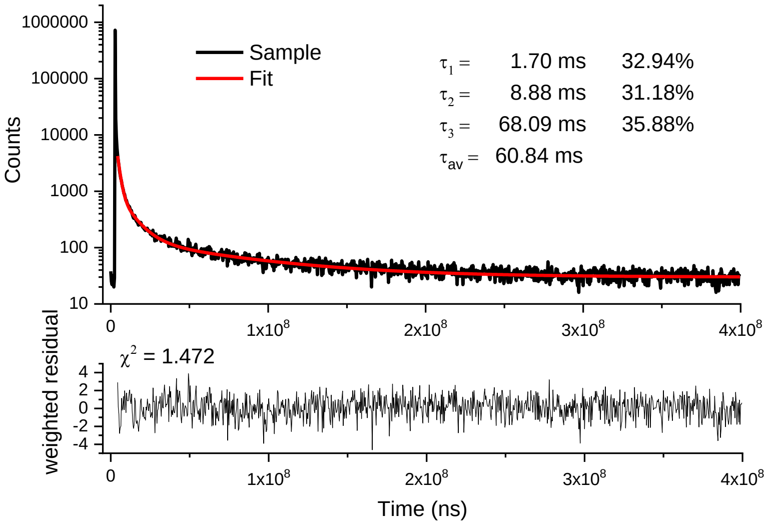

Figure S18. Lifetime measurement (λ_{exc} = 300 nm, λ_{em} = 530 nm) of **TT-HThio** ground crystals at 77 K.

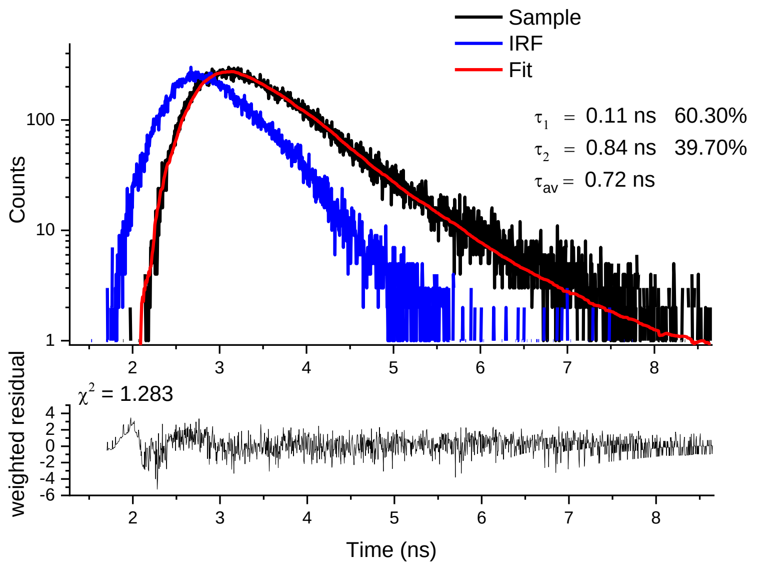

Figure S19. Lifetime measurement (λ_{exc} = 300 nm, λ_{em} = 365 nm) of **TT-HThio** in PMMA film 0.5% wt at 298 K.

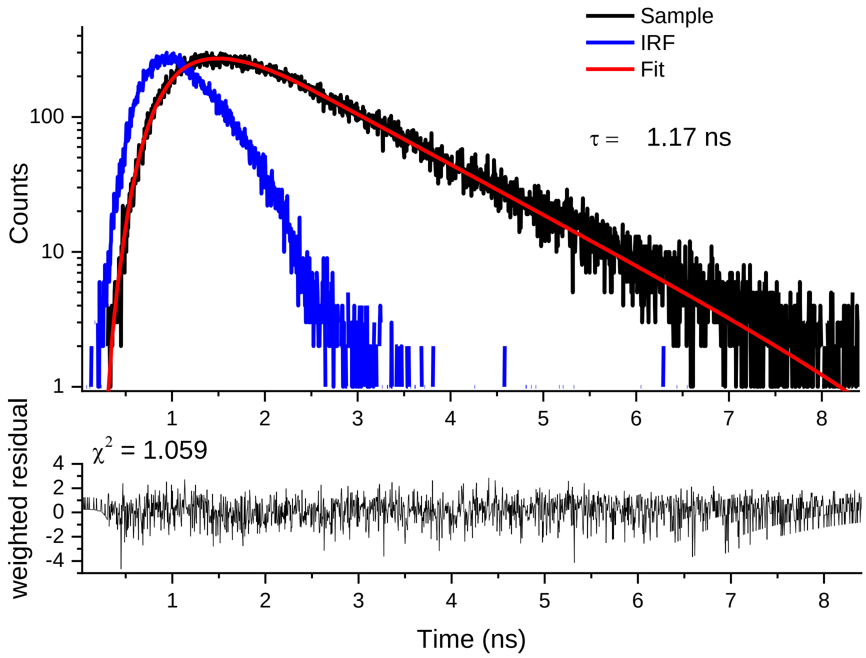

Figure S20. Lifetime measurement (λ_{exc} = 300 nm, λ_{em} = 380 nm) of **TT-(HThio)$_3$** in DCM 2·10^{-5} M at 298 K.

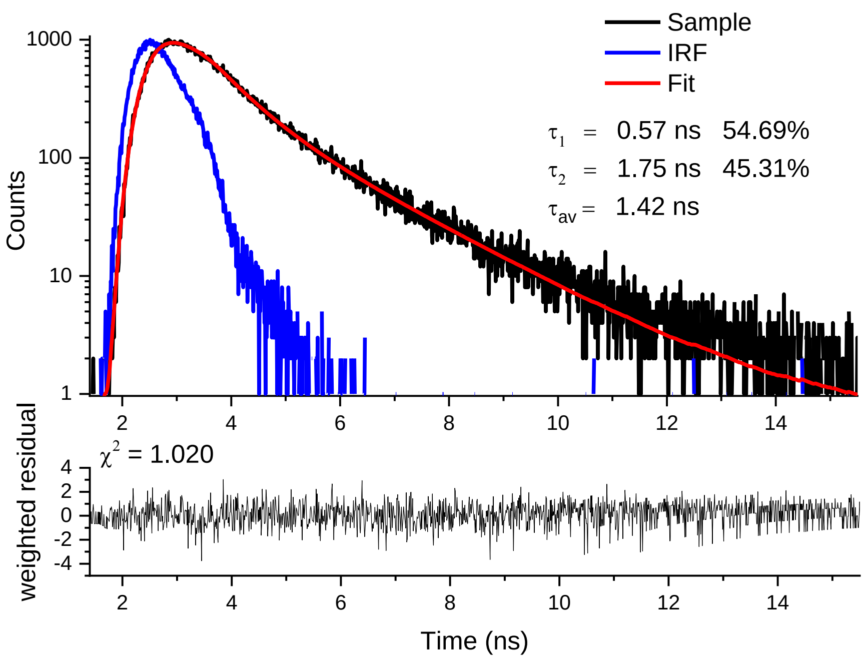

Figure S21. Lifetime measurement (λ_{exc} = 300 nm, λ_{em} = 380 nm) of **TT-(HThio)**$_3$ in DCM $2{\cdot}10^{-5}$ M at 77 K.

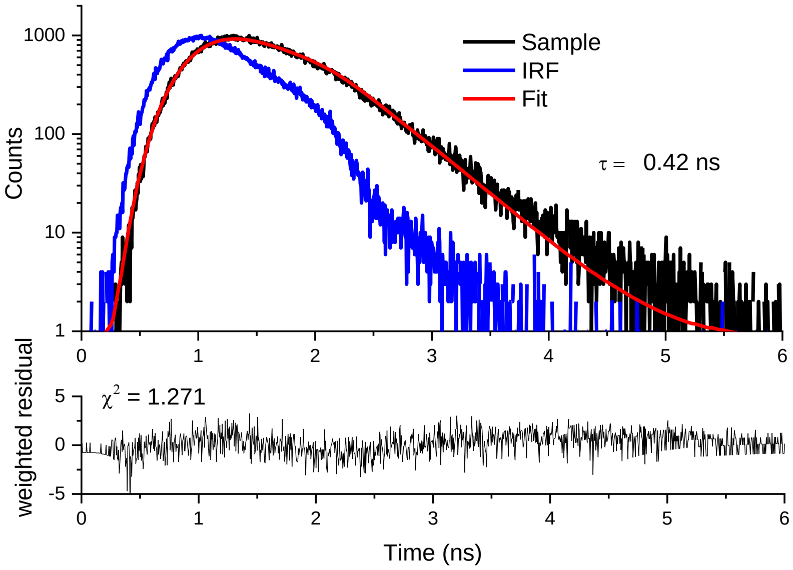

Figure S22. Lifetime measurement (λ_{exc} = 300 nm, λ_{em} = 382 nm) of crystalline powders of **TT-(HThio)**$_3$ at 298 K.

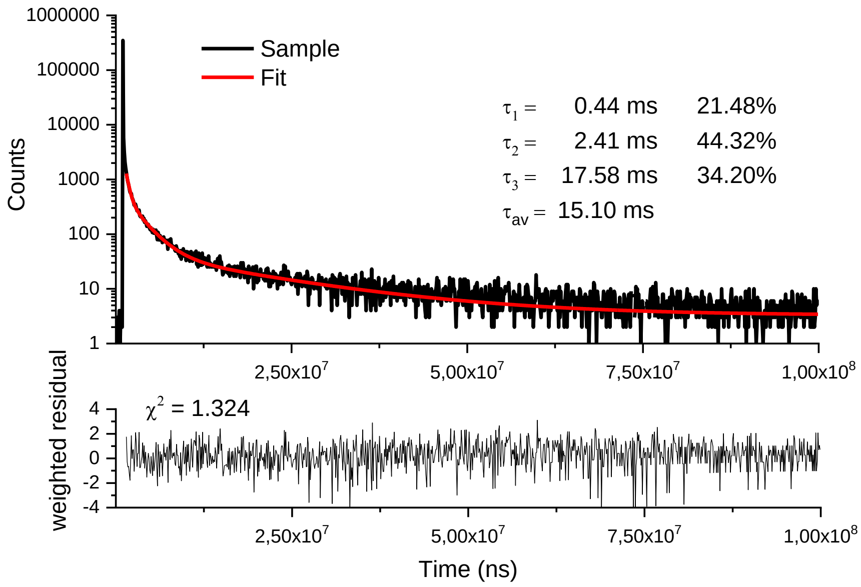

Figure S23. Lifetime measurement (λ_{exc} = 300 nm, λ_{em} = 452 nm) of crystalline powders of **TT-(HThio)**$_3$ at 298 K.

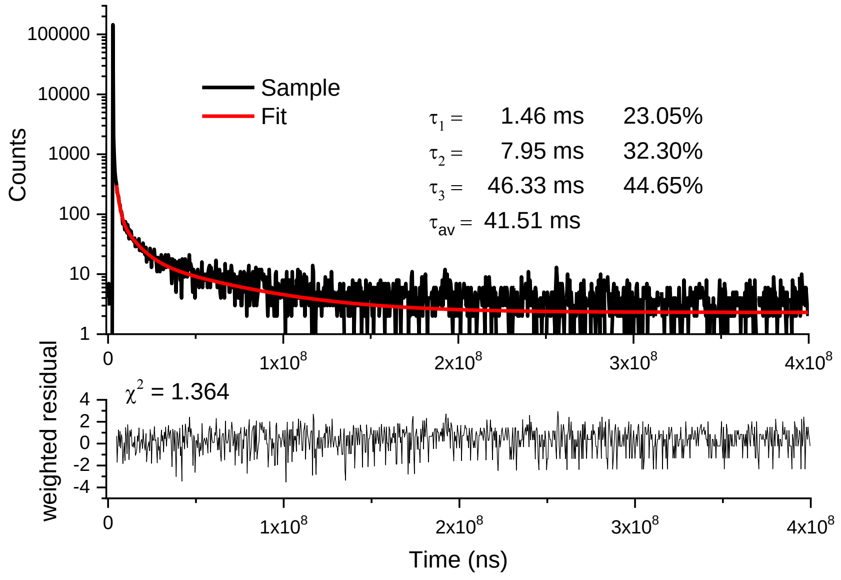

Figure S24. Lifetime measurement (λ_{exc} = 300 nm, λ_{em} = 514 nm) of crystalline powders of **TT-(HThio)**$_3$ at 298 K.

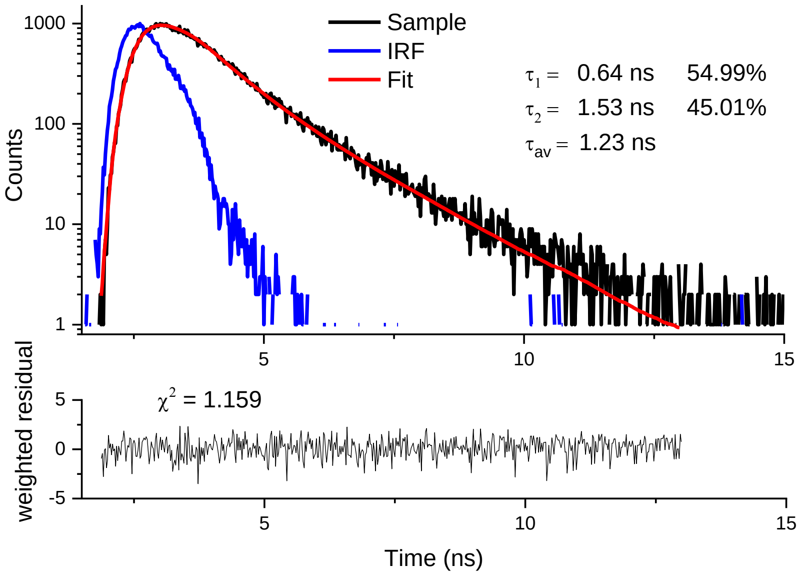

Figure S25. Lifetime measurement (λ_{exc} = 300 nm, λ_{em} = 365 nm) of crystalline powders of **TT-(HThio)**$_3$ at 77 K.

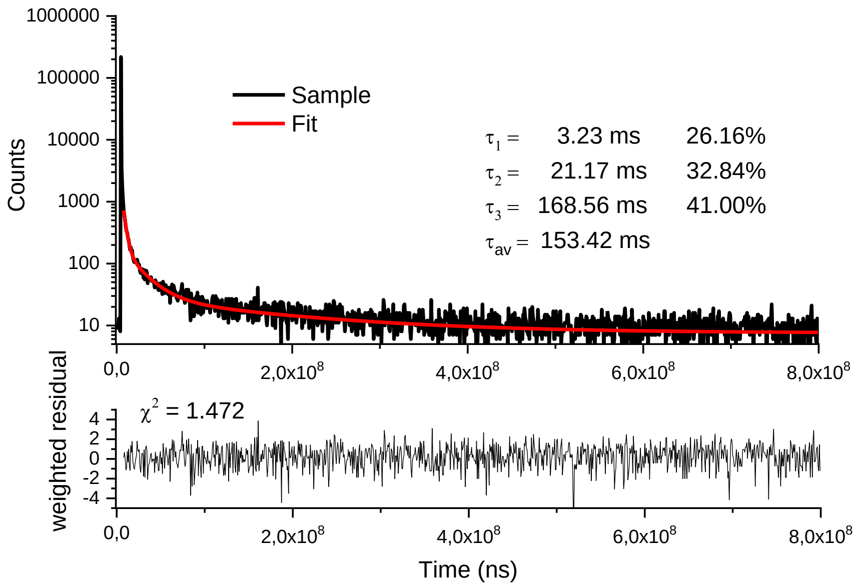

Figure S26. Lifetime measurement (λ_{exc} = 300 nm, λ_{em} = 423 nm) of crystalline powders of **TT-(HThio)**$_3$ at 77 K.

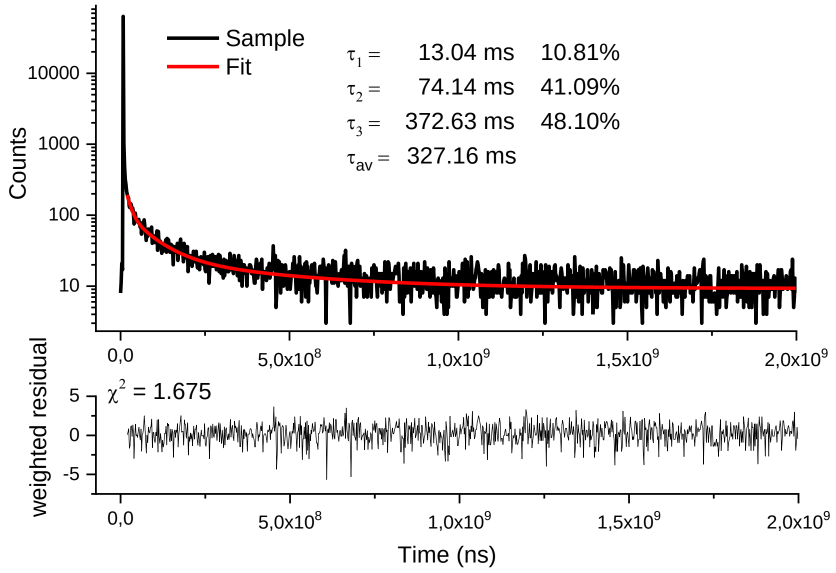

Figure S27. Lifetime measurement (λ_{exc} = 300 nm, λ_{em} = 513 nm) of crystalline powders of **TT-(HThio)**$_3$ at 77 K.

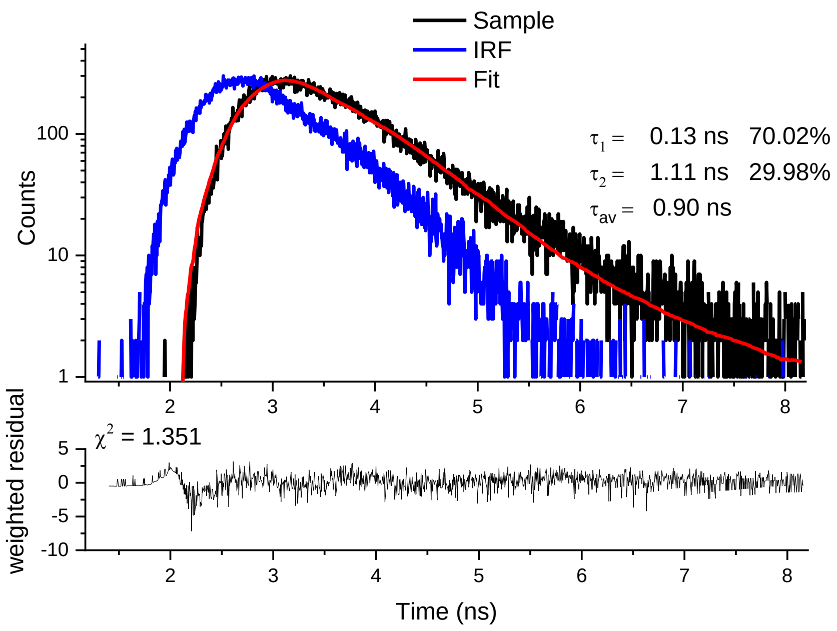

Figure S28. Lifetime measurement (λ_{exc} = 300 nm, λ_{em} = 372 nm) of of **TT-(HThio)**$_3$ in PMMA film 0.5% wt at 298 K.

4. AIE Tests

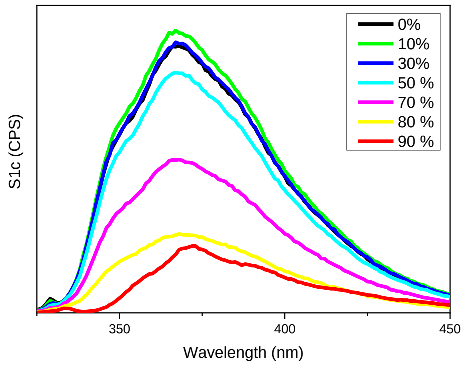

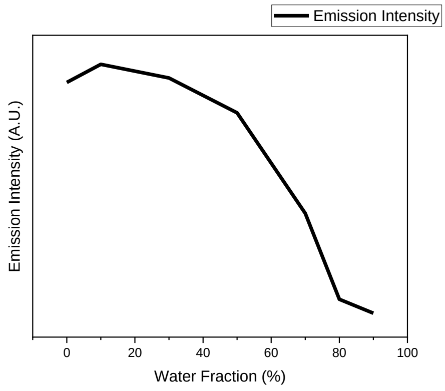

Figure S29. Left: Solvent-Non Solvent analysis THF/H_2O, Right: Plot of emission intensity vs Water fraction

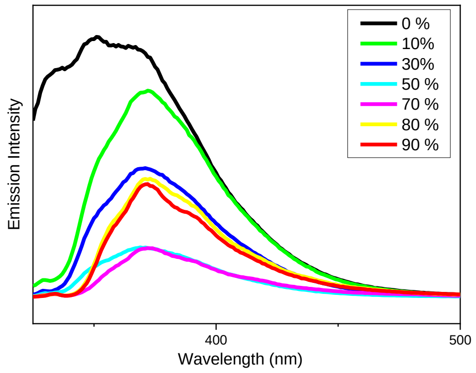

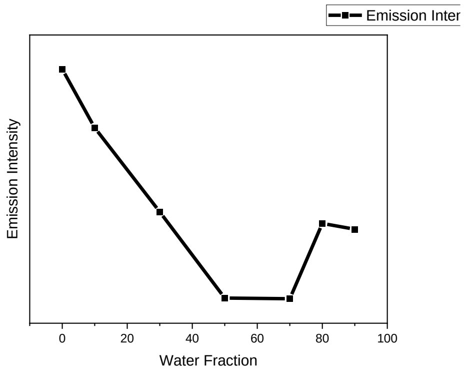

Figure S30. Left: Solvent-Non Solvent analysis DMSO/H_2O, Right: Plot of emission intensity vs Water fraction

5. Theoretical Studies

Table S2. First TD-ωB97X/6-311++G(d,p) triplet and singlet electronic transitions computed for **TT-HThio** at the optimized ground state geometry.

```
T1 Excited State   1:      Triplet-A      3.0045 eV  412.66 nm  f=nd      <S**2>=2.000
      92 ->106        0.13239
      93 -> 97        0.10276
      93 ->106        0.10118
      96 -> 97        0.59347
      96 ->106        0.18843
      96 <- 97        0.10086

 T2 Excited State   2:      Triplet-A      3.8624 eV  321.00 nm  f=nd      <S**2>=2.000
      92 -> 97        0.20283
      93 -> 97        0.25214
      94 -> 97        0.12515
      95 -> 99        0.36986
      96 -> 97       -0.14019
      96 ->105       -0.11762
      96 ->106        0.26418
      96 ->116       -0.12207

 T3 Excited State   3:      Triplet-A      4.0501 eV  306.13 nm  f=nd      <S**2>=2.000
      92 -> 97       -0.27804
      93 ->106       -0.14874
      94 -> 97       -0.11479
      95 -> 99        0.40652
      96 -> 97        0.13377
      96 ->106       -0.17809
      96 ->116        0.15517

 T4 Excited State   4:      Triplet-A      4.1123 eV  301.50 nm  f=nd      <S**2>=2.000
      92 -> 99       -0.14658
      93 -> 99        0.42483
      94 -> 99        0.10500
      95 -> 97        0.25331
      95 ->106       -0.24371
      95 ->116       -0.10951
      96 -> 99       -0.13337

 T5 Excited State   5:      Triplet-A      4.2749 eV  290.03 nm  f=nd      <S**2>=2.000
      92 -> 97       -0.12068
      93 -> 97       -0.16314
      94 -> 97        0.49753
      94 ->105       -0.12194
      94 ->106        0.31363
      94 ->116       -0.13023

 S1 Excited State   6:      Singlet-A      4.8045 eV  258.06 nm  f=0.4929  <S**2>=0.000
      96 -> 97        0.66731

 T6 Excited State   7:      Triplet-A      4.9062 eV  252.71 nm  f=nd      <S**2>=2.000
      92 -> 99       -0.21258
      92 ->106        0.16801
      95 -> 97        0.10790
      95 ->106       -0.11125
      96 -> 99        0.40532
      96 ->106       -0.17651
      96 ->116       -0.16845
      96 ->128        0.10969
      96 ->133       -0.10261

 T7 Excited State   8:      Triplet-A      5.1048 eV  242.88 nm  f=nd      <S**2>=2.000
      92 -> 99        0.12448
      93 -> 99       -0.26490
      93 ->116       -0.15083
      95 -> 97        0.36223
      95 ->106       -0.17427
      96 ->106        0.12004
```

```
 T8 Excited State   9:      Triplet-A      5.1542 eV  240.55 nm  f=nd      <S**2>=2.000
      86 -> 99         0.11208
      92 -> 97        -0.14545
      93 -> 97         0.27880
      93 -> 99        -0.10311
      93 ->106        -0.21120
      93 ->128        -0.10133
      95 -> 97        -0.13920
      95 -> 99        -0.19539
      95 ->116        -0.20559
      95 ->117         0.10719
      95 ->119        -0.13646

 S2 Excited State  10:      Singlet-A      5.4090 eV  229.22 nm  f=0.0282  <S**2>=0.000
      93 -> 97         0.22575
      93 -> 99         0.11845
      94 -> 97         0.15273
      95 -> 97        -0.25725
      95 -> 99         0.34081
      95 ->106         0.13369
      96 -> 99        -0.28501
      96 ->106         0.21721

 S3 Excited State  11:      Singlet-A      5.6221 eV  220.53 nm  f=0.0880  <S**2>=0.000
      93 -> 97        -0.18190
      94 -> 97         0.55918
      94 ->106         0.25422

 T9 Excited State  12:      Triplet-A      5.6634 eV  218.92 nm  f=nd      <S**2>=2.000
      82 -> 99        -0.15351
      92 -> 99        -0.15131
      92 ->106        -0.10287
      94 -> 97         0.12194
      95 ->128         0.10348
      96 -> 99         0.22896
      96 ->106         0.15154
      96 ->123         0.17875
      96 ->128        -0.12845

T10 Excited State  13:      Triplet-A      5.7366 eV  216.13 nm  f=nd      <S**2>=2.000
      96 ->103        -0.11344
      96 ->104         0.14711
      96 ->109         0.12071
      96 ->111         0.12796
      96 ->114        -0.16402
      96 ->117        -0.11967
      96 ->119         0.12961
      96 ->120        -0.17257
      96 ->127        -0.13868

 S4 Excited State  14:      Singlet-A      5.8005 eV  213.75 nm  f=0.0596  <S**2>=0.000
      93 -> 97         0.16853
      93 -> 99        -0.14276
      95 -> 97         0.17382
      95 -> 99         0.27959
      96 -> 99         0.38906
      96 ->105        -0.14210
      96 ->106         0.21564
      96 ->116        -0.15747

T11 Excited State  15:      Triplet-A      5.8083 eV  213.46 nm  f=nd      <S**2>=2.000
      92 -> 97        -0.11824
      94 -> 97        -0.11287
      94 ->109         0.12106
      94 ->110         0.10705
      94 ->114        -0.12850
      94 ->116        -0.15368
      94 ->117        -0.12462
      94 ->119         0.15366
      94 ->121         0.10627
      94 ->123        -0.10115
      96 ->105        -0.10567
      96 ->133         0.11419

 S5 Excited State  16:      Singlet-A      5.9656 eV  207.83 nm  f=0.1947  <S**2>=0.000
      95 -> 97         0.13671
```

```
      95 -> 99         0.38305
      96 ->103         0.11825
      96 ->105         0.20888
      96 ->106        -0.28629
      96 ->111        -0.10560
      96 ->114         0.12057
      96 ->116         0.13518

T12 Excited State  17:      Triplet-A      5.9757 eV  207.48 nm  f=nd      <S**2>=2.000
      92 -> 97         0.15101
      92 ->116         0.10218
      94 ->109         0.11547
      94 ->114        -0.14073
      94 ->116        -0.14426
      94 ->119         0.12421
      94 ->120        -0.10721
      96 ->105         0.13015
      96 ->109        -0.11976
      96 ->111        -0.11902
      96 ->114         0.10960
      96 ->116         0.14432
      96 ->119        -0.11207

 S6 Excited State  18:      Singlet-A      6.0190 eV  205.99 nm  f=0.0995  <S**2>=0.000
      92 -> 97         0.12863
      93 -> 99        -0.16623
      95 -> 99        -0.15903
      96 ->100        -0.12890
      96 ->104        -0.14979
      96 ->106         0.24220
      96 ->109        -0.14624
      96 ->110        -0.12312
      96 ->111        -0.18304
      96 ->114         0.13266
      96 ->116         0.10759
      96 ->117         0.14020
      96 ->119        -0.16087
      96 ->120         0.13178

 S7 Excited State  19:      Singlet-A      6.0408 eV  205.24 nm  f=0.4915  <S**2>=0.000
      92 -> 97         0.10230
      93 -> 99         0.27825
      95 -> 97         0.43823
      95 ->106        -0.17848
      96 -> 99        -0.24059
      96 ->106         0.16271

T13 Excited State  20:      Triplet-A      6.0427 eV  205.18 nm  f=nd      <S**2>=2.000
      86 -> 99        -0.14694
      92 -> 97        -0.11631
      92 -> 99        -0.12087
      93 -> 97        -0.15715
      93 -> 99         0.12022
      93 ->116        -0.13179
      95 -> 97        -0.21652
      95 ->106         0.10606
      95 ->124         0.10129
      95 ->128        -0.12204
      96 -> 99         0.23339
      96 ->106         0.19163
```

Table S3. First TD-ωB97X/6-311++G(d,p) triplet and singlet electronic transitions computed for **TT-(HThio)**$_3$ at the optimized ground state geometry.

```
T1 Excited State   1:      Triplet-A      2.9925 eV  414.32 nm  f=nd      <S**2>=2.000
     184 -> 189        0.19594
     184 -> 190       -0.22893
     184 -> 193       -0.11937
     185 -> 187       -0.17640
     185 -> 188        0.33478
     186 -> 187        0.33734
     186 -> 188        0.17661
```

```
T2 Excited State   2:      Triplet-A      3.0472 eV  406.88 nm  f=nd      <S**2>=2.000
    184 -> 187        0.33620
    185 -> 188       -0.25773
    186 -> 187        0.25494
    186 -> 189        0.19814
    186 -> 190       -0.23021
    186 -> 193       -0.11962

T3 Excited State   3:      Triplet-A      3.0476 eV  406.82 nm  f=nd      <S**2>=2.000
    184 -> 188        0.33678
    185 -> 187       -0.25599
    185 -> 189        0.19832
    185 -> 190       -0.23031
    185 -> 193       -0.11961
    186 -> 188       -0.25546

T4 Excited State   4:      Triplet-A      3.8278 eV  323.91 nm  f=nd      <S**2>=2.000
    179 -> 187        0.21444
    179 -> 188        0.17166
    180 -> 187        0.17168
    180 -> 188       -0.21456
    184 -> 190        0.10375
    185 -> 187       -0.11520
    185 -> 188        0.11595
    185 -> 206       -0.14349
    185 -> 207       -0.13295
    185 -> 209        0.13414
    186 -> 187        0.11577
    186 -> 188        0.11521
    186 -> 206       -0.13289
    186 -> 207        0.14357
    186 -> 210        0.13385

T5 Excited State   5:      Triplet-A      3.9827 eV  311.31 nm  f=nd      <S**2>=2.000
    178 -> 187        0.18148
    178 -> 188       -0.12568
    179 -> 187       -0.13739
    179 -> 188       -0.13541
    180 -> 187        0.13527
    180 -> 188       -0.13736
    180 -> 189       -0.10377
    180 -> 190        0.12331
    184 -> 207       -0.15639
    185 -> 188        0.13095
    185 -> 207       -0.12399
    185 -> 226       -0.10765
    186 -> 187       -0.13109
    186 -> 206        0.12400

T6 Excited State   6:      Triplet-A      3.9827 eV  311.31 nm  f=nd      <S**2>=2.000
    178 -> 187        0.12575
    178 -> 188        0.18146
    179 -> 187       -0.13544
    179 -> 188        0.13733
    179 -> 189       -0.10372
    179 -> 190        0.12334
    180 -> 187       -0.13739
    180 -> 188       -0.13527
    184 -> 206        0.15622
    185 -> 187        0.13114
    185 -> 206       -0.12415
    186 -> 188        0.13094
    186 -> 207       -0.12418
    186 -> 226        0.10772

T7 Excited State   7:      Triplet-A      4.2730 eV  290.16 nm  f=nd      <S**2>=2.000
    181 -> 189        0.22078
    181 -> 190       -0.26200
    181 -> 193       -0.13712
    182 -> 188       -0.30371
    182 -> 206       -0.15297
    182 -> 209        0.12686
    183 -> 187        0.31123
    183 -> 207       -0.15709
    183 -> 210       -0.12970
```

```
 T8 Excited State   8:      Triplet-A      4.2781 eV  289.81 nm  f=nd      <S**2>=2.000
     181 -> 187        0.30091
     181 -> 207       -0.15624
     181 -> 210       -0.12354
     182 -> 187       -0.15913
     182 -> 188        0.14705
     183 -> 187        0.14367
     183 -> 188        0.15604
     183 -> 189        0.21625
     183 -> 190       -0.25689
     183 -> 193       -0.13474

 T9 Excited State   9:      Triplet-A      4.2782 eV  289.80 nm  f=nd      <S**2>=2.000
     181 -> 188        0.30640
     181 -> 206        0.15904
     181 -> 209       -0.12614
     182 -> 187        0.14347
     182 -> 188        0.15785
     182 -> 189       -0.21635
     182 -> 190        0.25700
     182 -> 193        0.13472
     183 -> 187        0.15183
     183 -> 188       -0.14113

 S1 Excited State  10:      Singlet-A      4.8110 eV  257.71 nm  f=0.0046  <S**2>=0.000
     184 -> 189        0.15429
     184 -> 190       -0.16207
     185 -> 187       -0.22878
     185 -> 188        0.35954
     186 -> 187        0.40122
     186 -> 188        0.24013

T10 Excited State  11:      Triplet-A      4.8111 eV  257.71 nm  f=nd      <S**2>=2.000
     179 -> 188       -0.11775
     180 -> 187       -0.11760
     184 -> 226       -0.12760
     185 -> 187        0.27842
     185 -> 188        0.14140
     185 -> 207        0.11302
     186 -> 187        0.14123
     186 -> 188       -0.27874
     186 -> 206        0.11304

 S2 Excited State  12:      Singlet-A      4.8151 eV  257.49 nm  f=0.8566  <S**2>=0.000
     184 -> 187        0.38975
     184 -> 188        0.14984
     185 -> 188       -0.33235
     186 -> 187        0.28219
     186 -> 189        0.19523
     186 -> 190       -0.20121

 S3 Excited State  13:      Singlet-A      4.8155 eV  257.47 nm  f=0.8597  <S**2>=0.000
     184 -> 187       -0.15009
     184 -> 188        0.39141
     185 -> 187       -0.31264
     185 -> 189        0.19579
     185 -> 190       -0.20171
     186 -> 188       -0.30179

T11 Excited State  14:      Triplet-A      4.8969 eV  253.19 nm  f=nd      <S**2>=2.000
     178 -> 188       -0.17348
     184 -> 188        0.25340
     184 -> 206       -0.13160
     185 -> 187        0.15623
     186 -> 188        0.15585
     186 -> 226        0.11333

T12 Excited State  15:      Triplet-A      4.8971 eV  253.18 nm  f=nd      <S**2>=2.000
     178 -> 187       -0.17347
     184 -> 187        0.25383
     184 -> 207        0.13189
     185 -> 188        0.15586
     185 -> 226       -0.11340
     186 -> 187       -0.15562
```

```
 S4 Excited State  16:      Singlet-A      5.2714 eV  235.20 nm  f=0.0013  <S**2>=0.000
     179 -> 188        0.18589
     180 -> 187        0.18593
     184 -> 189       -0.14254
     184 -> 190        0.13304
     185 -> 187       -0.30804
     185 -> 188       -0.17419
     185 -> 207       -0.14028
     186 -> 187       -0.17413
     186 -> 188        0.30817
     186 -> 206       -0.14019

T13 Excited State  17:      Triplet-A      5.4925 eV  225.73 nm  f=nd      <S**2>=2.000
     157 -> 188        0.12347
     158 -> 187       -0.12346
     179 -> 188        0.12095
     180 -> 187        0.12084
     184 -> 189        0.10958
     184 -> 226       -0.12201
     185 -> 187       -0.14851
     185 -> 188       -0.11971
     186 -> 187       -0.11981
     186 -> 188        0.14876

 S5 Excited State  18:      Singlet-A      5.6170 eV  220.73 nm  f=0.1017  <S**2>=0.000
     181 -> 187        0.11076
     181 -> 189        0.20060
     181 -> 190       -0.21639
     181 -> 193       -0.11531
     182 -> 188       -0.24754
     183 -> 187        0.40759
     183 -> 207       -0.11830

 S6 Excited State  19:      Singlet-A      5.6173 eV  220.72 nm  f=0.0715  <S**2>=0.000
     181 -> 187        0.33292
     181 -> 207       -0.10356
     182 -> 187       -0.17101
     182 -> 188        0.26772
     183 -> 188        0.18224
     183 -> 189        0.21005
     183 -> 190       -0.22356
     183 -> 193       -0.11773

 S7 Excited State  20:      Singlet-A      5.6175 eV  220.71 nm  f=0.0688  <S**2>=0.000
     181 -> 188        0.35384
     181 -> 206        0.10828
     182 -> 187        0.17895
     182 -> 188        0.22168
     182 -> 189       -0.21945
     182 -> 190        0.23354
     182 -> 193        0.12303
     183 -> 187        0.12272
     183 -> 188       -0.16916

T14 Excited State  21:      Triplet-A      5.6838 eV  218.14 nm  f=nd      <S**2>=2.000
     184 -> 187       -0.10607
     184 -> 239        0.13726
     186 -> 189        0.13012
     186 -> 247       -0.10158

T15 Excited State  22:      Triplet-A      5.6840 eV  218.13 nm  f=nd      <S**2>=2.000
     184 -> 188       -0.10599
     184 -> 240       -0.13731
     185 -> 189        0.13017
     185 -> 247       -0.10164

T16 Excited State  23:      Triplet-A      5.7280 eV  216.45 nm  f=nd      <S**2>=2.000
     184 -> 211       -0.10058
     184 -> 235        0.11971
     184 -> 247       -0.16785

 S8 Excited State  24:      Singlet-A      5.7488 eV  215.67 nm  f=0.0062  <S**2>=0.000
     179 -> 187        0.19198
     180 -> 188       -0.19174
     184 -> 189       -0.16625
     184 -> 190        0.17590
```

```
     185 -> 187        0.12239
     185 -> 188        0.11956
     185 -> 206       -0.20564
     185 -> 207       -0.10884
     185 -> 209        0.15730
     186 -> 187        0.12025
     186 -> 188       -0.12219
     186 -> 206       -0.10922
     186 -> 207        0.20616
     186 -> 210        0.15725

T17 Excited State  25:      Triplet-A      5.7562 eV  215.39 nm  f=nd      <S**2>=2.000
     184 -> 224        0.10026
     184 -> 225       -0.10083
     184 -> 229       -0.10207
     185 -> 202       -0.10324
     185 -> 208       -0.11892
     185 -> 211       -0.12556
     185 -> 226        0.10068

T18 Excited State  26:      Triplet-A      5.7563 eV  215.39 nm  f=nd      <S**2>=2.000
     184 -> 224        0.10056
     184 -> 225        0.10021
     184 -> 230        0.10173
     186 -> 202       -0.10328
     186 -> 208       -0.11905
     186 -> 211       -0.12587
     186 -> 226        0.10086

T19 Excited State  27:      Triplet-A      5.8117 eV  213.34 nm  f=nd      <S**2>=2.000
     181 -> 208        0.11373
     181 -> 226       -0.12173
     182 -> 224        0.10298
     182 -> 227       -0.10446
     182 -> 229       -0.10121
     183 -> 225       -0.10320
     183 -> 228        0.10451
     183 -> 230       -0.10114

 S9 Excited State  28:      Singlet-A      5.8294 eV  212.69 nm  f=0.1570  <S**2>=0.000
     179 -> 188        0.12667
     180 -> 187       -0.12681
     184 -> 188        0.17003
     185 -> 187        0.26848
     185 -> 189        0.21720
     185 -> 190       -0.15574
     185 -> 207        0.15981
     185 -> 210        0.10447
     186 -> 188        0.26864
     186 -> 206       -0.15946
     186 -> 209        0.10464

S10 Excited State  29:      Singlet-A      5.8295 eV  212.68 nm  f=0.1574  <S**2>=0.000
     179 -> 187       -0.12658
     180 -> 188       -0.12703
     184 -> 187        0.17028
     185 -> 188        0.26834
     185 -> 206       -0.15996
     185 -> 209        0.10493
     186 -> 187       -0.26792
     186 -> 189        0.21745
     186 -> 190       -0.15563
     186 -> 207       -0.15974
     186 -> 210       -0.10452

T20 Excited State  30:      Triplet-A      5.8863 eV  210.63 nm  f=nd      <S**2>=2.000
     184 -> 187        0.13024
     184 -> 188       -0.17520
     185 -> 187       -0.13925
     186 -> 188       -0.13932

T21 Excited State  31:      Triplet-A      5.8864 eV  210.63 nm  f=nd      <S**2>=2.000
     184 -> 187       -0.17545
     184 -> 188       -0.13051
     185 -> 188       -0.13882
     186 -> 187        0.13890
```

```
S11 Excited State  32:      Singlet-A      5.9283 eV  209.14 nm  f=0.0709  <S**2>=0.000
     184 -> 187        0.12588
     184 -> 188        0.21354
     184 -> 206       -0.18366
     185 -> 202        0.11994
     185 -> 208        0.11307
     185 -> 211        0.13155
     185 -> 226       -0.12594
     186 -> 189       -0.19019

S12 Excited State  33:      Singlet-A      5.9284 eV  209.14 nm  f=0.0708  <S**2>=0.000
     184 -> 187        0.21346
     184 -> 188       -0.12556
     184 -> 207        0.18369
     185 -> 189        0.19024
     186 -> 202        0.11992
     186 -> 208        0.11240
     186 -> 211        0.13169
     186 -> 226       -0.12609
```
